# IDN2 and Its Paralogs Form a Complex Required for RNA–Directed DNA Methylation

**DOI:** 10.1371/journal.pgen.1002693

**Published:** 2012-05-03

**Authors:** Cui-Jun Zhang, Yong-Qiang Ning, Su-Wei Zhang, Qing Chen, Chang-Rong Shao, Yan-Wu Guo, Jin-Xing Zhou, Lin Li, She Chen, Xin-Jian He

**Affiliations:** National Institute of Biological Sciences, Beijing, China; University of Cambridge, United Kingdom

## Abstract

IDN2/RDM12 has been previously identified as a component of the RNA–directed DNA methylation (RdDM) machinery in *Arabidopsis thaliana*, but how it functions in RdDM remains unknown. By affinity purification of IDN2, we co-purified two IDN2 paralogs IDP1 and IDP2 (IDN2 PARALOG 1 and 2). The coiled-coil domain between the XS and XH domains of IDN2 is essential for IDN2 homodimerization, whereas the IDN2 C-terminal XH domain but not the coiled-coil domain is required for IDN2 interaction with IDP1 and IDP2. By introducing the wild-type *IDN2* sequence and its mutated derivatives into the *idn2* mutant for complementation testing, we demonstrated that the previously uncharacterized IDN2 XH domain is required for the IDN2-IDP1/IDP2 complex formation as well as for IDN2 function. IDP1 is required for *de novo* DNA methylation, siRNA accumulation, and transcriptional gene silencing, whereas IDP2 has partially overlapping roles with IDP1. Unlike IDN2, IDP1 and IDP2 are incapable of binding double-stranded RNA, suggesting that the roles of IDP1 and IDP2 are different from those of IDN2 in the IDN2-IDP1/IDP2 complex and that IDP1 and IDP2 are essential for the functioning of the complex in RdDM.

## Author Summary

In eukaryotes, transposable elements and other DNA repeats are important parts of genomes. Suppression of these sequences is required for genome stability and integrity. DNA methylation is an important chromatin modification that prevents the expression and movement of these repeat sequences. Small interfering RNAs initiate DNA methylation and transcriptional gene silencing of the sequences. In the plant model organism *Arabidopsis thaliana*, DNA methylation is mediated by an RNA–directed DNA methylation (RdDM) pathway. Here we report that the double-stranded RNA-binding protein IDN2 and it paralogs IDP1 and IDP2 (IDN2 PARALOG 1 and 2) cooperate and form a novel complex that is required for RNA–directed DNA methylation. Unlike IDN2, IDP1 and IDP2 have no double-stranded RNA binding ability. We propose that the IDN2-IDP1/2 is a complex that functions at a downstream step of the RdDM pathway. The findings significantly increase our understanding of the plant RdDM pathway as well as of the RNA–mediated chromatin changes in yeast and animals.

## Introduction

DNA methylation is an important epigenetic modification in eukaryotes including plants and animals. In *Arabidopsis thaliana*, DNA methylation mainly occurs in gene-coding regions, transposons, and other DNA repeats [1]–[3]. DNA methylation is maintained by DNA METHYLTRANSFERASE 1 (MET1) at CG sites and by CHROMOMETHYLASE 3 (CMT3) at CHG (H is A, T, or C) sites [4]–[6]. The SWI2/SNF2-like chromatin-remodeling protein DIFFICIENT IN DNA METHYLATION 1 (DDM1) is involved in the maintenance of DNA methylation [7]. The histone H3K9 methyltransferase KYP/SUVH4 cooperates with CMT3 and couples the regulation of DNA methylation and histone H3K9 dimethylation [8], [9]. Establishment of DNA methylation in *Arabidopsis* depends on an RNA-directed DNA methylation (RdDM) pathway [1], [3], [10]–[12].

In the past several years, the components of the RdDM pathway have been identified and characterized. The DOMAIN REARRANGED METHYLTRANSFERASE 2 (DRM2) catalyzes *de novo* DNA methylation in the RdDM pathway [13]–[16]. The DRM2 paralog DRM3 is also required for the full functioning of DRM2 [17]. Small interfering RNA (siRNA) and long noncoding RNA are required for the RdDM pathway [1], [3], [18], [19]. Two atypical plant-specific multi-subunit DNA-dependent RNA polymerases Pol IV and Pol V are required for biogenesis of siRNA and long noncoding RNA, respectively [11], [19]–[24]. Moreover, Pol II has a residual role in the RdDM pathway [25]. NUCLEAR RNA POLYMERASE D1 (NRPD1) is the largest subunits of Pol IV, whereas NUCLEAR RNA POLYMERASE E1 (NRPE1) is the largest subunit of Pol V [22]. Some subunits are present in one specific RNA polymerase, but some others are shared by Pol IV, Pol V, and/or Pol II [22]–[24]. Single-stranded RNA transcripts produced by Pol IV are converted into double-stranded RNAs by RNA-DEPENDENT RNA POLYMERASE 2 (RDR2) and cleaved into 24-nt siRNAs by DICER-LIKE 3 (DCL3) [10]. 3′-terminals of siRNAs are methylated at the 2′hydroxyl group and are stabilized by HUA ENHANCER 1 (HEN1) [26]. SiRNAs are loaded into ARGONAUTE proteins AGO4, AGO6, or AGO9, and assembled into RNA-induced transcriptional silencing (RITS) complexes that mediate *de novo* DNA methylation [27]–[31]. NRPE1 and KOW-CONTAINING TRANSCRIPTION FACTOR 1 (KTF1), a SPT5-like protein, contain conserved WG/GW repeats in their C-terminal domains, which interact with ARGONAUTE proteins and help form RITS complexes [32]–[34]. Pol V-dependent RNA transcripts are bound by AGO4 and KTF1 *in vivo* and act as scaffolds that recruit RITS complexes to specific chromatin regions [19]. Recruitment of KTF1 and AGO4 to chromatin is in parallel and independent. The interaction between KTF1 and AGO4 on chromatin seems to create a platform that facilitates recruitment of the *de novo* DNA methyltansferase DRM2 [35].

DEFECTIVE IN RNA-DIRECTED DNA METHYLATION 1 (DRD1), a chromatin-remodeling protein, DEFECTIVE IN MERISTEM SILENCING 3 (DMS3), a protein containing a hinge domain of structural maintenance of chromosome (SMC) proteins, and RNA-DIRECTED DNA METHYLATION 1 (RDM1), a methylated DNA-binding protein, form a tight protein complex DDR (DRD1, DMS3, and RDM1) that is required for producing Pol V-dependent RNA transcripts [36]–[39]. RDM1 is also capable of binding AGO4 and DRM2, suggesting that RDM1 may be involved in recruitment of AGO4-containing RITS complexes to DRM2 [38]. RNA-DIRECTED DNA METHYLATION 4 (RDM4)/DEFECTIVE IN MERISTEM SILENCING 4 (DMS4) is a transcription factor that interacts with Pol II, Pol IV and Pol V [40]–[42]. Disruption of *RDM4* independently affects accumulation of Pol V-dependent RNA transcripts and Pol II-dependent protein-coding genes [40], [41]. The RDM4 homolog IWR1 in yeast is involved in Pol II assembly in the cytoplasm and directs the transportation of the fully assembled Pol II into the nucleus [24]. Based on this study, it is likely that RDM4 may facilitate the assembly and nuclear import of Pol II, Pol IV, and Pol V. CLASSY 1 (CLSY1), a chromatin-remodeling protein, and SAWADEE HOMEODOMAIN HOMOLOG 1 (SHH1)/DNA-BINDING TRANSCRIPTION FACTOR 1 (DTF1) are required for *de novo* DNA methylation and accumulation of 24-nt siRNAs [42]–[44]. Affinity purification of Pol IV co-purified RDR2, CLSY1, and SHH1/DTF1, indicating that CLSY1 and SHH1 function in the upstream of the RdDM pathway and are involved in generation of Pol IV-dependent siRNAs [42]. However, CLSY1 and SHH1 affect siRNA accumulation at a subset of RdDM targets [42]–[44], suggesting CLSY1 and SHH1 probably interact with Pol IV in a chromatin locus-specific manner.

INVOLVED IN DE NOVO 2 (IDN2)/RNA-DIRECTED DNA METHYLATION 12 (RDM12) was independently identified as an RdDM component by two forward genetic screening systems [45], [46]. IDN2 contains an N-terminal C2H2-type zinc finger domain, an XS domain, a coiled-coil domain, and an XH domain [45]. In *Arabidopsis*, IDN2 is a member of a protein family that contains eight other IDN2-like proteins. Additionally, IDN2 is similar to SUPPRESSOR OF GENE SILENCING 3 (SGS3), an important component in post-transcriptional gene silencing pathways [47], [48]. Both IDN2 and SGS3 contain zinc finger, XS, and coiled-coil domains. The XS domains are required for IDN2 and SGS3 to bind 5′ overhanging double-stranded RNA [45], [49]. SGS3 is involved in accumulation of viral siRNAs, ta-siRNAs, and nat-siRNAs [48], [50], [51]. However, disruption of IDN2 only partially affects the accumulation of heterochromatic siRNAs from a subset of RdDM target loci, suggesting that IDN2 may function at a downstream step of the RdDM pathway [45], [46]. The coiled-coil domain is sufficient for dimerization of many proteins [52]. It is possible that the IDN2 coiled-coil domain may help IDN2 to form a homodimer with itself or a heterodimer with other members of the IDN2 family in *Arabidopsis*. The XH domain is highly conserved in the IDN2 family but not in SGS3 [47], indicating that the XH domain may be required for the functional specificity of the IDN2 protein family.

In the present study we show that IDN2 associates with two IDN2 paralogs in the IDN2 protein family, which were named IDP1 and IDP2 (after IDN2 PARALOG 1 and 2). IDP1 is required for siRNA accumulation, *de novo* DNA methylation, and transcriptional gene silencing, whereas IDP2 has partially overlapping roles with IDP1. The IDN2 coiled-coil domain is essential for the homodimerization of IDN2 with itself but is not required for IDN2 association with IDP1 and IDP2. The uncharacterized XH domain of IDN2 is required for association with IDP1 and IDP2 but not for IDN2 homodimerization. IDN2 and IDP1 or IDP2 (IDP1/IDP2) form an IDN2-IDP1/IDP2 complex through the IDN2 coiled-coil domain and XH domain. Unlike IDN2, IDP1 and IDP2 are incapable of binding double-stranded RNA, suggesting that IDP1 and IDP2 have distinct roles in the IDN2-IDP1/IDP2 complex. The IDN2-IDP1/IDP2 complex may facilitate the recruitment of double-stranded RNA-containing effector complexes to specific chromatin regions at a downstream step of the RdDM pathway.

## Results

### IDN2 associates with itself and two IDN2 paralogs, IDP1 and IDP2

**Figure 1:**
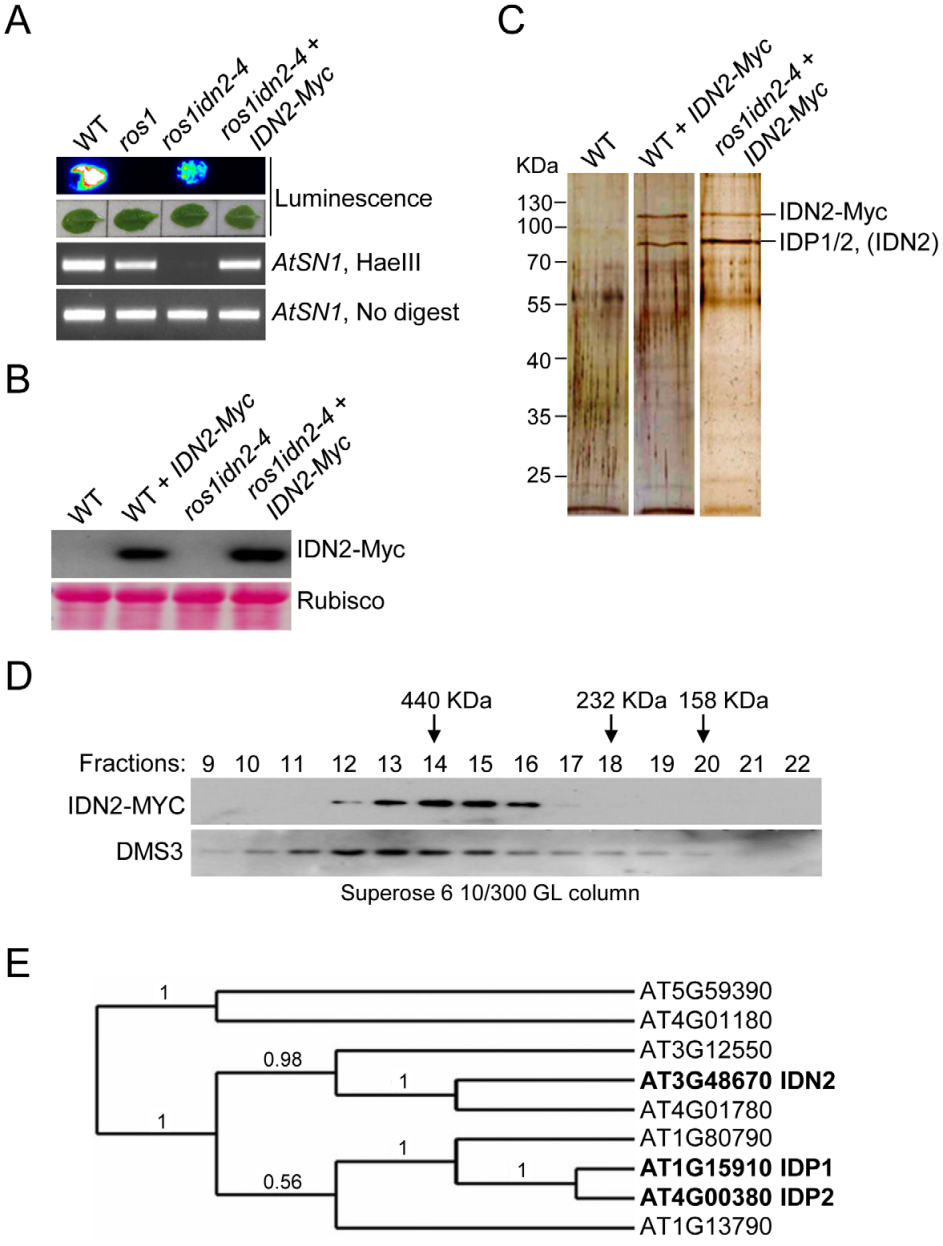
Characterization of the IDN2 complex. (A) The *IDN2-6xMyc* transgene restored the silencing of *RD29A-LUC* transgene in *ros1idn2-4* as well as the DNA methylation of *AtSN1*. The leaves were collected for luminescence imaging after treatment with 2% NaCl for 6 h. *AtSN1* methylation was tested by chop-PCR. (B) Western blot analysis of the *IDN2-6xMyc* transgene expression by the Myc antibody in wild type and *ros1idn2-4*. Ponceau S staining of Rubisco is shown as a loading control. (C) Protein extracts from the *IDN2-6xMyc* transgenic plants in wild type and *ros1idn2-4* were affinity purified by the Myc antibody. Purified proteins were separated by SDS-PAGE gel and visualized by silver staining. KDa, Kilodalton. (D) Western blot analysis of the elution profiles of IDN2-6xMyc in *ros1idn2-4* by the Myc antibody. Fraction numbers and sizes of standard proteins are shown. The elution profile of the previously characterized ∼400 to 500-KDa DMS3 complex is indicated as a control for gel filtration. (E) The phylogenetic tree for IDN2 and eight IDN2-like proteins in *Arabidopsis*. IDN2 and the IDN2-interacting proteins AT1G15910 (IDP1) and AT4G00380 (IDP2) are marked in black.

To understand how IDN2 functions in the RdDM pathway, we generated a construct harboring a native *IDN2* promoter-driven *IDN2-6xMyc* fusion transgene and transformed it into either wild-type or *ros1idn2-4* mutant plants. Previous studies had shown that the silencing of the *RD29A* promoter-driven luciferase transgene (*RD29A-LUC*) in *ros1* was partially released by mutation of *IDN2/RDM12* in the *ros1idn2* mutant [46]. Our results revealed that the *IDN2-6xMyc* construct was able to restore the silencing of the *RD29A* promoter-driven luciferase transgene and complement the DNA methylation defect of *AtSN1* in *ros1idn2-4*, demonstrating that the IDN2-6xMyc fusion protein is functional *in vivo* (Figure 1A). The *IDN2-6xMyc* expression in the transgenic lines was confirmed by western blotting with the Myc antibody (Figure 1B).

The *IDN2-6xMyc* transgenic lines in the wild-type and *ros1idn2-4* backgrounds were used for affinity purification of IDN2-6xMyc, respectively. The co-purified proteins from both wild-type and *ros1idn2-4* backgrounds contained two major bands with one at ∼110 KDa and another at ∼80 KDa (Figure 1C). Mass-spectrometric assay was carried out to identify co-purified proteins. The ∼110-KDa band was the IDN2-6xMyc fusion protein in both wild-type and *ros1idn2-4* backgrounds. The ∼80-KDa band from the wild-type background was rich in the IDN2 paralog 1 (IDP1) AT1G15910 and to a lesser extent the IDN2 paralog 2 (IDP2) AT4G00380 and IDN2 itself, whereas the band from the *ros1idn2-4* background was composed of the two IDN2 paralogs but not IDN2 itself (Table 1, Table S1). The results suggest that IDN2 is capable of interact with its paralogs IDP1 and IDP2 *in vivo*. Moreover, IDN2 was detected in the ∼80-KDa co-purified proteins from the wild-type background but not from the *ros1idn2-4* mutant background, suggesting that the endogenous IDN2 protein in the wild-type background was co-purified by affinity purification of IDN2-6xMyc. Taken together, IDN2 not only interacts with its two paralogs IDP1 and IDP2, but also interacts with itself *in vivo*.

**Table 1:** Mass-spectrometric analysis of IDN2-6xMyc affinity purification.

Accession number	Protein	Mascot score	MW (Da)	^1^Matched queries	^2^Matched peptides	^3^Unique matches
Total IDN2-Myc purified proteins in WT						
IPI00524938	AT3G48670 IDN2	2341	74778	118	45	118
IPI00523067	AT1G15910 IDP1	1480	72590	68	37	53
IPI00542085	AT4G00380 IDP2	444	73119	34	24	19
The ∼80 KDa band from IDN2-Myc purified proteins in WT						
IPI00523067	AT1G15910 IDP1	2092	72590	135	54	88
IPI00542085	AT4G00380 IDP2	612	73119	59	30	12
IPI00524938	AT3G48670 IDN2	528	74778	33	26	33
The ∼80 KDa band from IDN2-Myc purified proteins in *ros1idn2*						
IPI00523067	AT1G15910 IDP1	1891	72590	76	35	46
IPI00542085	AT4G00380 IDP2	606	73119	37	20	7
The ∼80 KDa band from IDN2E600Q-Myc purified proteins in *ros1idn2*						
IPI00523067	AT1G15910 IDP1	829	72590	26	21	20
IPI00542085	AT4G00380 IDP2	208	73119	10	10	4
The ∼80 KDa band from IDN2W616A-Myc purified proteins in *ros1idn2*						
IPI00523067	AT1G15910 IDP1	458	72590	25	15	19
IPI00542085	AT4G00380 IDP2	143	73119	10	8	4

The protein extracts were prepared from the *IDN2-6xMyc* transgenic plants in the wild-type and *ros1idn2-4* mutant backgrounds and used for affinity purification of the IDN2-6xMyc-containing complex. Either total purified proteins or the ∼80-KDa band were subjected to mass-spectrometric analysis. Mascot score of each protein is shown. 1) The number is the total number of mass spectra matched to the corresponding protein, including the redundant ones that match to the same peptides. 2) The number is deduced from the “Matched queries” by removing the redundant peptides. 3) The number is calculated from “Matched queries” by removing the overlapped peptide sequences among its homologous protein family.

We further prepared the protein extracts from the *IDN2-6xMyc* transgenic lines for gel filtration followed by western blotting with the Myc antibody. The elution peak of the IDN2-6xMyc-containing complex was at ∼400 KDa, whereas no other peak was found by western blotting (Figure 1D). The result demonstrates that all IDN2 proteins exist in a ∼400-KDa protein complex, in agreement with the tetramer size of the IDN2-IDP1/2 complex in the *IDN2-6xMyc* transgenic plants. The ∼400 to 500-KDa elution peak for the previously demonstrated DMS3-containing complex is shown as a control of the gel filtration (Figure 1D).

Beside IDN2, the IDN2 protein family contains eight IDN2-like proteins including At3g12550, At4g01780, At1g15910, At4g00380, At1g13790, At1g80790, At5g59390, and At4g01180. IDN2 is most similar to At3g12550 and At4g01780, which belong to the same clade as IDN2 according to sequence alignment and phylogenetic analysis (Figure S1 and Figure 1E). IDP1 (AT1G15910) and IDP2 (AT4G00380) are highly similar to each other and exist in another clade of the family (Figure S1, Figure S2 and Figure 1E). However, IDP1 and IDP2 but not At3g12550 or At4g01780 were co-purified by affinity purification of IDN2. The online *Arabidopsis* microarray database shows that all the genes in the *IDN2* family are well expressed, indicating that the IDN2 interaction specificity with IDP1 and IDP2 cannot be attributed to the different expression of the genes in the *IDN2* family.

### Characterization of the IDN2-IDP1/2 interaction by yeast two-hybrid

**Figure 2:**
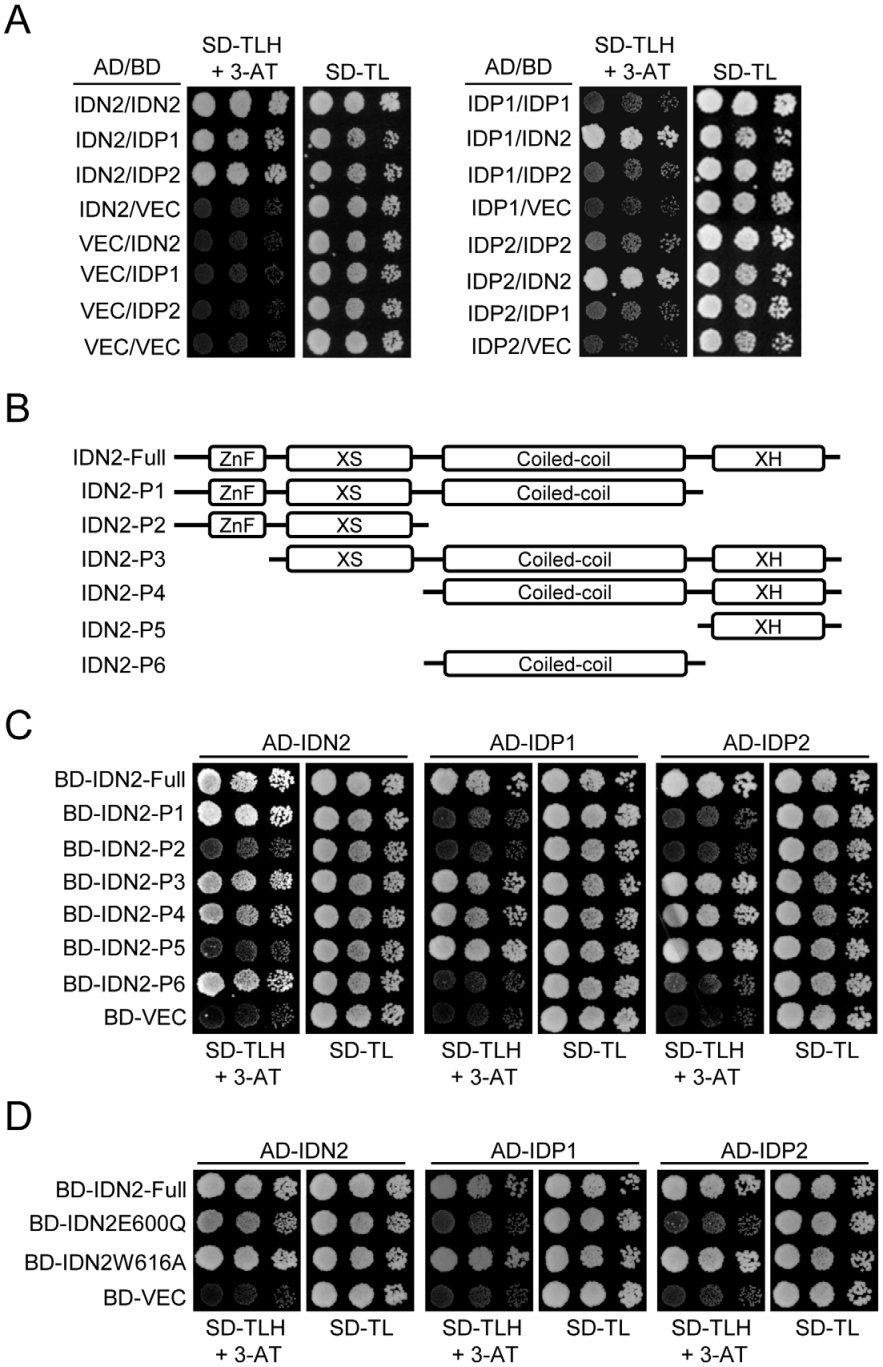
Identification of the IDN2 domains required for interaction with IDN2, IDP1, and IDP2 by yeast two-hybrid analysis. (A) Interaction of IDN2, IDP1 and IDP2 by yeast two-hybrid analysis. *IDN2*, *IDP1* and *IDP2* were separately cloned into pGADT7 and pGBKT7 vectors. The constructs were cotransformed into yeast strain PJ694a. Each transformed yeast strain was assayed on the plates with the indicated mediums. Columns in each panel represent serial decimal dilutions. SD, synthetic dropout medium. VEC represents the empty pGADT7 or pGBKT7 vector. (B) Diagram of the full-length IDN2 protein and its truncated versions used in yeast two-hybrid analysis. The zinc finger domain, XS domain, coiled-coil domain, and XH domain are indicated. (C) Interaction between truncated IDN2 proteins and IDN2, IDP1 or IDP2 in yeast two-hybrid analysis. The truncated *IDN2* sequences were separately cloned into the pGBKT7 vector and cotransformed with pGADT7 constructs harboring IDN2, IDP1 or IDP2. The transformed strains were used for yeast two-hybrid analysis as above. BD-VEC represents the empty pGBKT7 vector. (D) Interaction between the mutated full-length IDN2 and the wild-type IDN2, IDP1 or IDP2 in yeast two-hybrid analysis. The mutations are located in the IDN2 XH domain.

Yeast two-hybrid assay was carried out to further characterize the IDN2 interaction with itself and its paralogs IDP1 and IDP2. The results showed that IDN2 was capable of directly interacting with itself as well as with its paralogs IDP1 and IDP2. The interaction occured whenever IDN2 was used as a bait or prey (Figure 2A). Interestingly, IDP1 and IDP2 were unable to dimerize by themselves or to interact with each other (Figure 2A), indicating that IDN2 may have some critical characteristics that are absent in IDP1 and IDP2 and that are required for dimerization.

To identify the IDN2 domain that is responsible for IDN2 interaction, we generated a series of truncated IDN2 constructs in bait vectors for yeast two-hybrid assay (Figure 2B). According to this assay, all the constructs containing the IDN2 coiled-coil domain were capable of forming a homodimer with IDN2, whereas the constructs without the coiled-coil domain were incapable of forming a homodimer with IDN2 (Figure 2C). This suggests that the IDN2 coiled-coil domain is sufficient for the homodimerization of IDN2, which is consistent with the expected role of the conserved coiled-coil domain. Unexpectedly, the IDN2 coiled-coil domain was not required for association with IDP1 and IDP2 (Figure 2C), although the full length of IDN2 including the coiled-coil domain shows similarity with IDP1 and IDP2 (Figure S1 and Figure S2). Interestingly, the previously uncharacterized IDN2 XH domain was required for IDN2 association with IDP1 as well as with IDP2 (Figure 2C). The XH domain by itself was sufficient for the association (Figure 2C). The results suggest that the IDN2 paralogs IDP1 and IDP2 are unlikely to be functionally redundant with IDN2.

Two point mutations were introduced in the IDN2 XH domain to validate the function of the XH domain in the IDN2-IDP1/2 interaction. The conserved glutamic acid 600 and tryptophan 616 in the XH domain was mutated to glutamine and alanine (IDN2E600Q and IDN2W616A), respectively. According to yeast two-hybrid assay, the IDN2E600Q mutation disrupted the interaction of IDN2 with its paralogs IDP1 and IDP2 but did not affect the dimerization of IDN2 with itself (Figure 2D). This confirms that the IDN2 XH domain is indeed essential for the interaction between IDN2 and IDP1/2, which may be required for the function of IDN2 *in vivo*. Additionally, the IDN2W616A mutation did not affect IDN2 interaction (Figure 2D).

### The IDN2 XH domain is required for the IDN2-IDP1/2 interaction and RdDM *in vivo*

**Figure 3:**
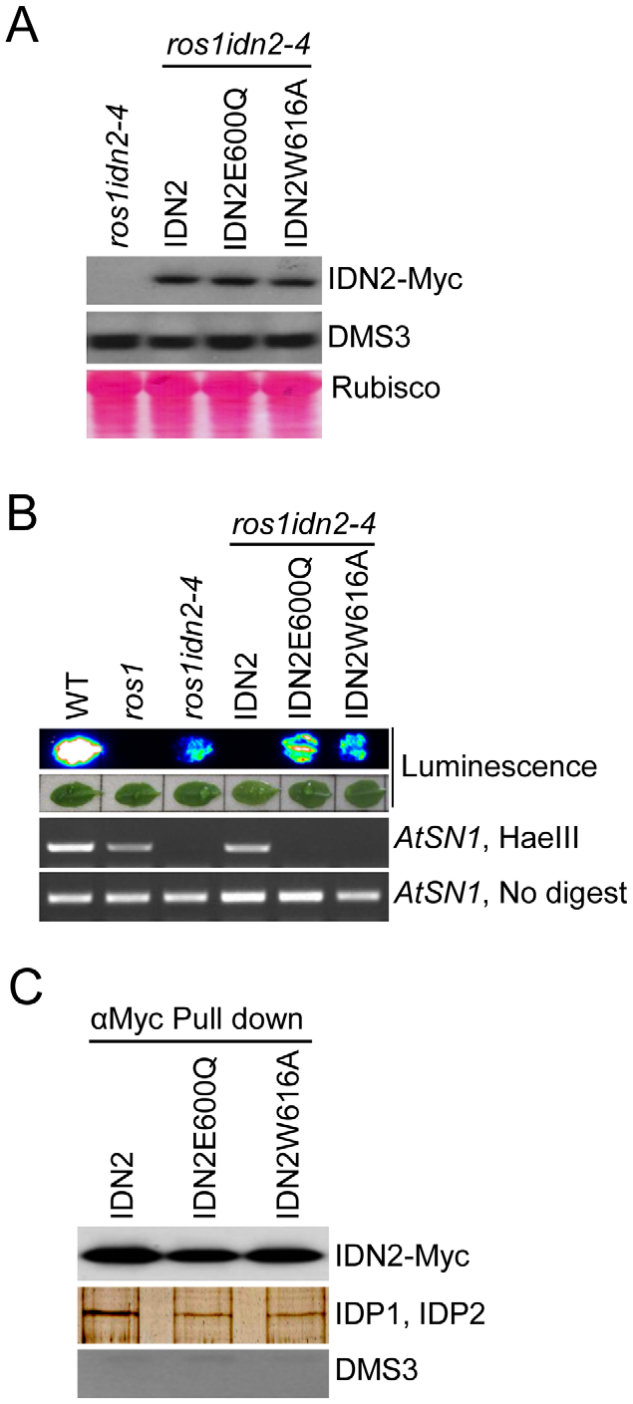
Effect of the mutations in the IDN2 XH domains on the IDN2 complex in *Arabidopsis*. (A) Western blotting of IDN2-6xMyc and DMS3 proteins in the wild-type *IDN2-6xMyc* and mutated *IDN2E600Q-6xMyc* and *IDN2W616A-6xMyc* transgenic plants in *ros1idn2-4*. The Ponceau S-stained Rubisco is shown as a loading control. (B) The *IDN2E600Q-6xMyc* and *IDN2W616A-6xMyc* transgenes in *ros1idn2-4* fail to restore the RD29A-LUC transgene silencing and complement the DNA methylation defects at *AtSN1*. (C) The effect of the IDN2E600Q and IDN2W616A mutations on the interaction between IDN2 and IDP1 or IDP2 was determined by pull-down assay. Affinity purification with the Myc antibody was applied for the protein extracts from the wild-type *IDN2-6xMyc*, mutated *IDN2E600Q-6xMyc* and mutated *IDN2W616A-6xMyc* transgenic plants in *ros1idn2-4*. Co-purified proteins were tested by western blotting using the Myc antibody and endogenous DMS3 antibody. The purified proteins were also separated on SDS-PAGE and visualized by silver staining. The ∼80-KDa bands are shown.

To investigate the function of the IDN2 XH domain *in vivo*, we introduced the IDN2E600Q and IDN2W616A mutations into the functional wild-type *IDN2-6xMyc* fusion construct and transformed them into the *ros1idn2-4* double mutant for complementation testing. Western blotting showed that the expression of both mutated IDN2 fusion proteins was comparable to that of wild-type IDN2-6xMyc (Figure 3A). The accumulation of DMS3, a component of the DDR complex in RdDM, was not affected by the *idn2* mutation in *ros1idn2-4* (Figure 3A). While the wild-type *IDN2-6xMyc* transgenic lines complemented the silencing defect of the *RD29A-LUC* transgene in the *ros1idn2-4* mutant background, the *IDN2E600Q-6xMyc* and *IDN2W616A-6xMyc* transgenic lines were unable to restore the silencing of the *RD29A-LUC* transgene (Figure 3B). Moreover, the two mutations also caused the mutated *IDN2* transgenes to fail to complement the DNA methylation defect of *idn2* at a canonical RdDM target *AtSN1* (Figure 3B). These results demonstrate that the IDN2 XH domain is essential for the function of IDN2 in RdDM and in transcriptional gene silencing *in vivo*. The IDN2E600Q mutation not only disrupted the interaction between IDN2 and IDP1/2 (Figure 2D), but also reduced the function of IDN2 *in vivo* (Figure 3B), suggesting that the association of IDN2 with IDP1/2 through the XH domain is required for the RdDM pathway. In contrast, the IDN2W616A mutation affected the IDN2 function in RdDM (Figure 3B) but it did not disrupt the IDN2-IDP1/2 interaction in yeast two-hybrid assay (Figure 2D).

To test the potential role of the IDN2 XH domain in the IDN2-IDP1/2 interaction in *vivo*, we affinity purified the IDN2-6xMyc complex from the wild-type *IDN2-6xMyc* transgenic plants, as well as from the mutated *IDN2E600Q-6xMyc* and *IDN2W616A-6xMyc* transgenic plants in the *ros1idn2-4* background. Each purified complex was run on SDS-PAGE and was subjected to western blotting and mass-spectrometric analysis. Western blotting showed that the IDN2-6xMyc fusion protein was equivalently affinity purified from the wild-type and two mutated *IDN2-6xMyc* transgenic plants (Figure 3C). The co-purified ∼80-KDa band was visualized from all the three purified protein samples on the silver-staining gel. Mass-spectrometric analysis indicated that the ∼80-KDa band from all the three samples comprised both IDP1 and IDP2 (Table 1, Table S1). However, on the silver-staining gel, the band from affinity purification of wild-type IDN2-6xMyc was much stronger than that from purification of mutated IDN2E600Q-6xMyc or IDN2W616A-6xMyc (Figure 3C), suggesting that both IDN2E600Q and IDN2W616A mutations reduce IDN2 interaction with IDP1 and IDP2 *in vivo*. No DMS3 protein was detectable in the IDN2-6xMyc purified proteins (Figure 3C), indicating the absence of interaction between IDN2 and DMS3.

### IDP1 is required for DNA methylation and has overlapping roles with IDP2

As IDN2 paralogs in the IDN2-IDP1/2 complex, IDP1 and IDP2 may be required for RdDM. To investigate the potential roles of IDP1 and IDP2 in RdDM, we used the homozygous T-DNA insertion mutants *idp1-1* (SALK_075378) and *idp2-1* (SALK_066712) for analysis (Figure S3A, S3B). The gene-specific primers flanking T-DNA insertion sites were used to detect the RNA transcripts of *IDP1* and *IDP2* in their T-DNA mutant lines by RT-PCR. The *IDP1* and *IDP2* RNA transcripts were undetectable in the *idp1-1* and *idp2-1* T-DNA mutant lines (Figure S3C), suggesting that the T-DNAs disrupt the function of these genes.

**Figure 4:**
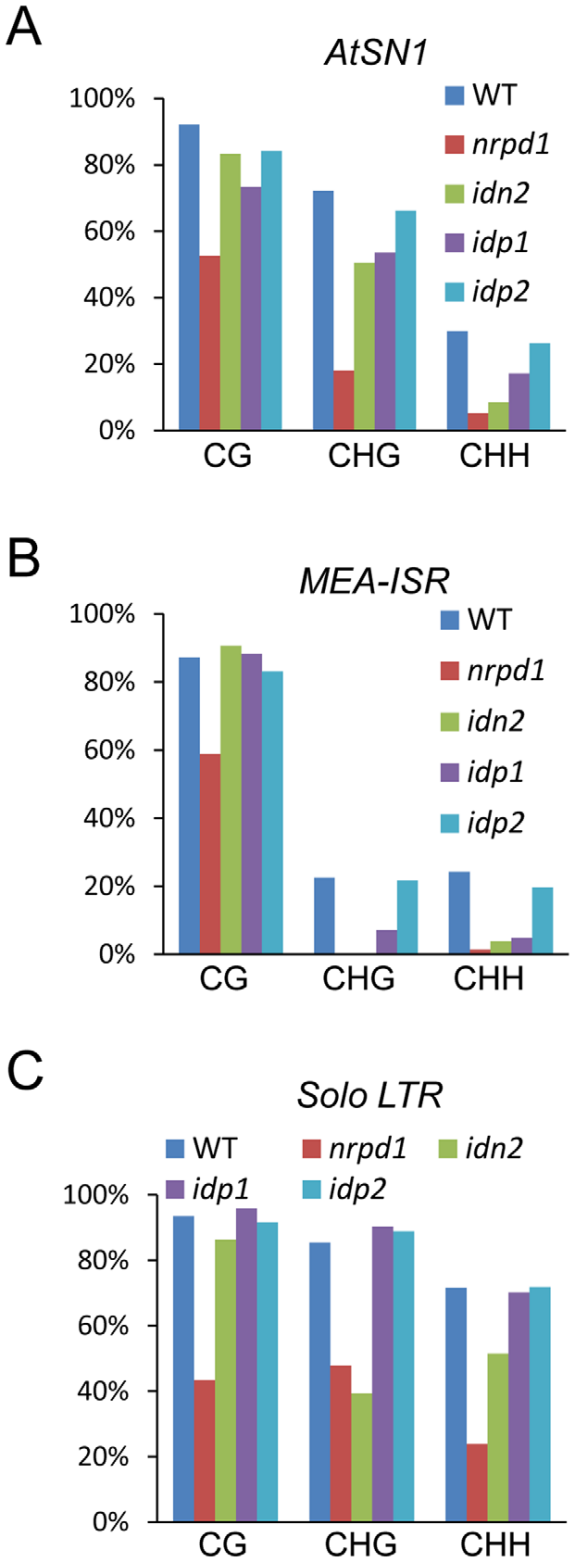
Effect of *idp1* and *idp2* on DNA methylation. (A–C) Effect of *idp1* and *idp2* on the DNA methylation of *AtSN1*, *MEA-ISR*, and *Solo LTR* was determined by bisulfite sequencing. The percentage of methylated cytosine in different cytosine contexts is shown. The DNA methylation in each genotype is shown by different colored bar as indicated.

The DNA methylation level was assessed in the *idn2-5*, *idp1* and *idp2* T-DNA mutants at several well-characterized RdDM targets, including *AtSN1*, *IGN5*, *AtMU1*, *MEA-ISR*, and *Solo LTR*. By digestion with the methylation-sensitive enzyme HaeIII, followed by PCR, we found that the DNA methylation of *AtSN1* and *IGN5* was reduced in *idn2-5* and *idp1* but not in *idp2* (Figure S4A). The reductions of DNA methylation in *idn2-5* and *idp1* were comparable, but were less than that in *nrpd1*. Southern blotting showed that the *AtMU1* methylation was reduced in *idp1* as well as in *nrpd1* but not in *idp2* (Figure S4B). Bisulfite sequencing showed that the DNA methylation levels of *AtSN1* and *MEA-ISR* were reduced in *nrpd1*, *idn2-5* and *idp1*, particularly at CHG and CHH sites (Figure 4A, 4B and Figures S5, S6). The effect of *idn2-5* and *idp1* on the DNA methylation of *AtSN1* and *MEA-ISR* was less than that of *nrpd1*. The DNA methylation level of *Solo LTR* was markedly reduced in *nrpd1* and partially reduced in *idn2-5*, but did not change in *idp1* (Figure 4C and Figure S7).

To confirm that *IDP1* is the right gene required for RdDM, we produced a construct harboring the wild-type *IDP1* genomic sequence fused in frame with a 3xFlag encoding sequence (*IDP1-3xFlag*) and transformed it into *idp1* for a complementation assay. The western blotting result showed that the *IDP1-3xFlag* transgene was properly expressed in the *idp1* mutant (Figure S8A). The DNA methylation analysis result indicated that *IDP1-3xFlag* transgene complemented the DNA methlyation defect of *idp1* at the canonical RdDM target *AtSN1* (Figure S8B). As an IDN2 paralog, IDP1 is also required for DNA methylation.

The *idp2* mutation had no significant effect on DNA methylation at all tested RdDM target loci (Figure 4 and Figure S4). However, in the *idp1idp2* double mutant, the DNA methylation levels of *AtSN1* and *IGN5* were highly reduced compared to the *idp1* single mutants (Figure S9), suggesting that IDP1 and IDP2 had overlapping roles in regulation of DNA methylation.

### IDP1 is required for the silencing of RdDM targets and the accumulation of 24-nt siRNAs

**Figure 5:**
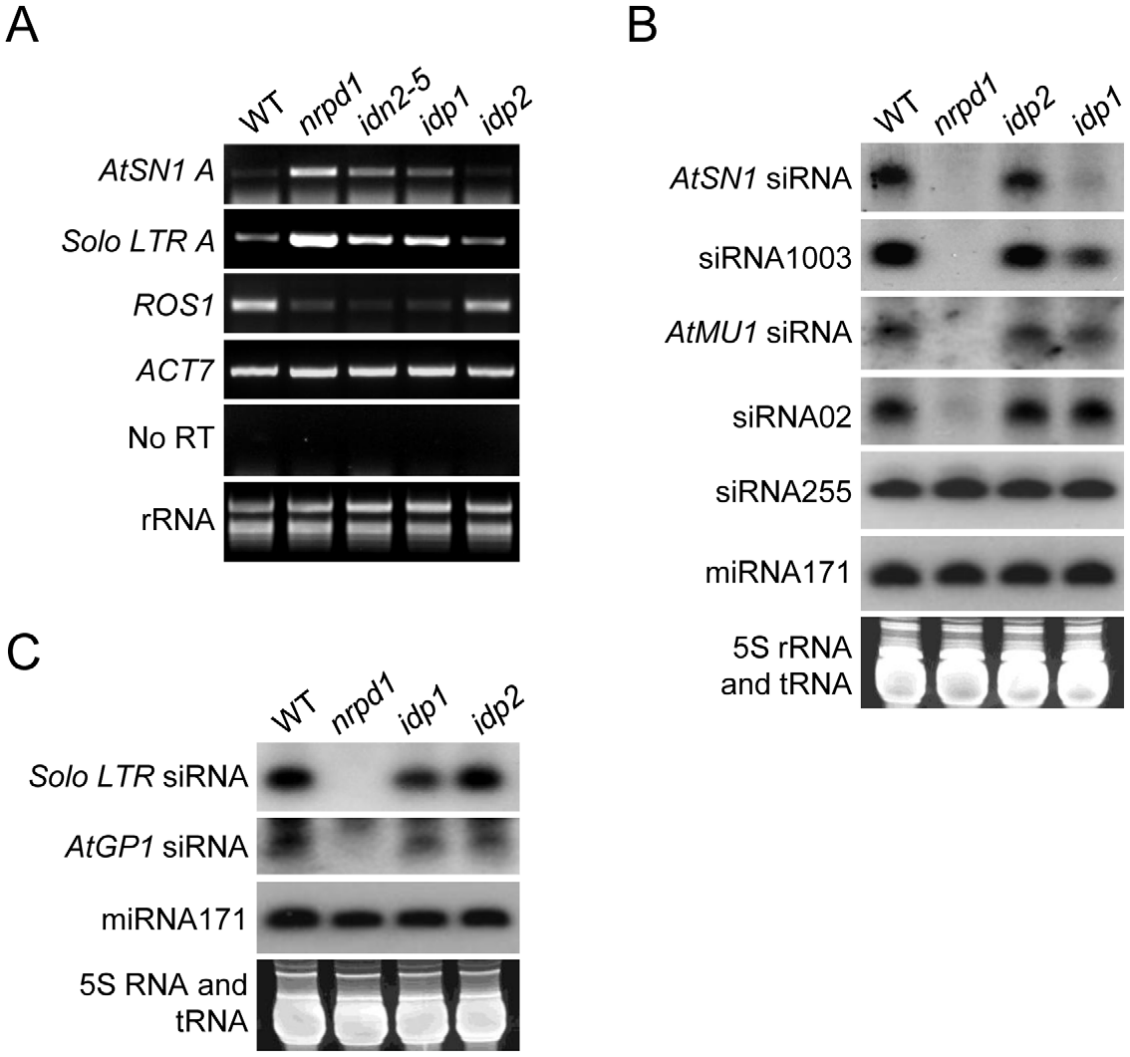
Effect of *idp1* and *idp2* on transcriptional gene silencing and small RNA accumulation at endogenous RdDM target loci. (A) Effect of *idp1* and *idp2* on transcriptional gene silencing of endogenous RdDM targets was determined by semiquantitative RT-PCR. *ACT7* was used as an internal control. No RT amplification shows the absence of DNA contamination in RNA samples. (B, C) Effect of *idp1* and *idp2* on small RNA accumulation was determined by small RNA northern blotting. 24-nt siRNAs, 21-nt ta-siRNA255, and miRNA171 were detected. The ethidium bromide-stained small RNA gel was imaged as a loading control.

We tested whether the reduction of DNA methylation in *idp1* results in elevated transcript level at RdDM target loci by semiqantitative RT-PCR. The results showed that the transcripts of *AtSN1* and *Solo LTR* were enhanced in *nrpd1* and to a lesser extent in *idn2-5* and *idp1* (Figure 5A). As expected, *idp2* had no effect on the transcripts of the RdDM target loci (Figure 5A). The results suggest that the reduction of DNA methylation caused by *idp1* leads to the release of transcriptional gene silencing. Interestingly, although the *Solo LTR* DNA methylation level was not changed in *idp1*, its RNA transcript was markedly increased. *Solo LTR* was previously characterized as a specific RdDM target that is regulated by RdDM in DNA methylation-dependent and –independent manners [44]. Like the previously identified SHH1/DTF1 [42], [44], IDP1 is required for the silencing of *Solo LTR* in a DNA methylation-independent manner. Previous studies demonstrated that the *ROS1* RNA transcript level was reduced by the disruption of MET1, DDM1 and the RdDM machinery that affected genomic DNA methylation [40], [53], [54]. Our results show that the *ROS1* transcript level is reduced in *idp1*, *idn2-5*, and *nrpd1* but not in *idp2*, supporting the idea that IDP1 is an RdDM component that associates with IDN2 (Figure 5A).

We carried out small RNA blot analysis to determine whether the *idp1* mutation affected the accumulation of 24-nt siRNAs from RdDM target loci. The results revealed that *idp1* partially reduced the accumulation of *AtSN1* siRNA, siRNA1003, and *Solo LTR* siRNA, whereas it had no effect on the other tested siRNAs including *AtMU1* siRNA, *AtGP1* siRNA, and siRNA02 (Figure 5B, 5C). The accumulation level of ta-siRNA255 and miRNA171 was not affected by *idp1* and *nrpd1*. As expected, the *idp2* mutation did not significantly affect the accumulation of 24-nt siRNAs at RdDM targets (Figure 5B, 5C). The results suggest that as an IDN2-interacting paralog, IDP1 specifically affects the accumulation of 24-nt siRNAs at a subset of RdDM targets including *AtSN1* siRNA, siRNA1003, and *Solo LTR* siRNA.

Previous studies demonstrated that the accumulation of *AtSN1* siRNA, siRNA1003, and *Solo LTR* siRNA are dependent on both Pol IV and Pol V, whereas *AtMU1* siRNA, siRNA02, and *AtGP1* siRNA are Pol IV-dependent but Pol V-independent [33], [40], [44]. Our small RNA northern blotting results indicated that the *idp1* mutation had no effect on the siRNAs that are Pol IV-dependent but Pol V-independent, whereas it partially reduced the siRNAs that are dependent on both Pol IV and Pol V. The results support that IDP1 does not directly initiate siRNA generation but affect siRNA accumulation in the same manner with Pol V and several other downstream components of RdDM. IDP1 probably acts at a downstream step in the RdDM pathway, which is consistent with the possible role of IDN2 in the RdDM pathway [45].

### IDN2, but not IDP1 and IDP2, binds double-stranded RNA

**Figure 6:**
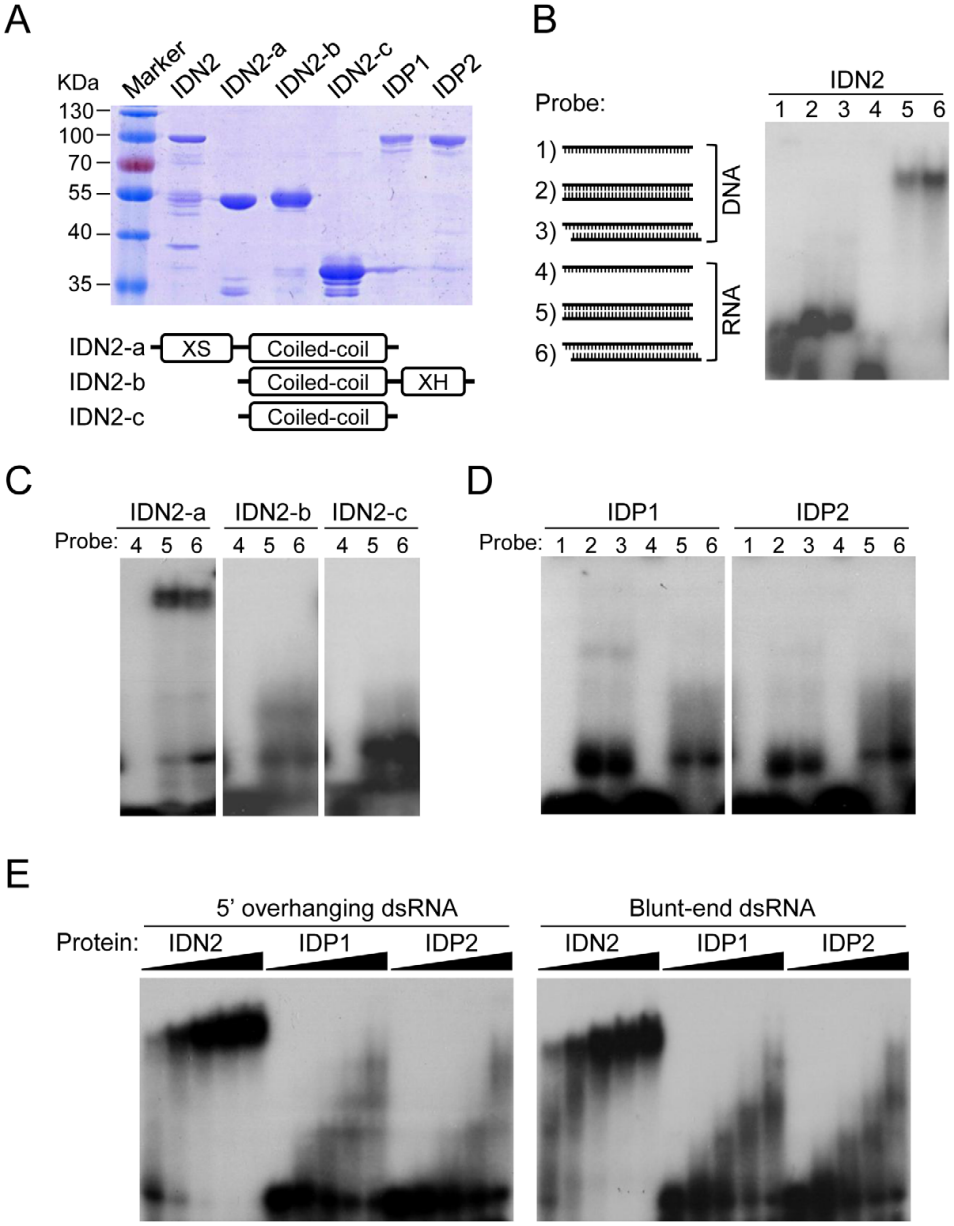
IDN2, but not IDP1 or IDP2, binds double-stranded RNA. (A) The bacterially expressed, full-length and truncated IDN2 proteins and full-length IDP1 and IDP2 proteins were purified and subjected to SDS-PAGE. The gel was stained with Coomassie. The bottom panel shows the diagram of the truncated IDN2 proteins. (B) The diagrams of different probes (1–6) used in this study are shown. The sizes of two 5′ overhangs of the 5′ overhanging double-stranded RNA are 2 nt. The single-stranded DNA and RNA probes are RD29A-DNA-F and RD29A-RNA-F (Table S4), respectively. The full-length IDN2 protein binds to 35-nt double-stranded RNA (corresponding to the RD29A promoter) with or without 5′ overhangs in EMSA. (C) IDN2-a, but not IDN2-b or IDN2-c, binds to double-stranded RNA. (D) Neither IDP1 nor IDP2 binds to double-stranded RNA or to the other types of nucleic acids. (E) EMSA was carried out when an increasing amount of indicated proteins (0.1, 0.4, 1.6, 3.2, 6.4 µg) was added in the RNA-binding system. 5′ overhanging double-stranded RNA and blunt-end double-stranded RNA were used in the binding assay, respectively.

IDN2 had been demonstrated to be an RNA-binding protein that specifically binds 5′ overhanging double-stranded RNA [45]. The truncated IDN2 proteins including the XS domain and coiled-coil domain are sufficient for the RNA-binding ability of IDN2 (Figure 6A) [45]. Because of the sequence similarity between IDN2 and its paralogs IDP1 and IDP2, it is reasonable to predict that IDP1 and IDP2 may also be capable of binding double-stranded RNA. For electrophoretic mobility shift assay (EMSA), the full-length IDN2, IDP1 and IDP2 proteins, and three truncated IDN2 proteins were expressed and purified from bacteria (Figure 6A). In our EMSA experiments, six different nucleic acid species were used as probes (Figure 6B). These probes were well labeled and purified (Figure S10). Our EMSA results indicated that the full-length IDN2 not only bound 5′ overhanging double-stranded RNA but also bound the double-stranded RNA with blunt ends (Figure 6B). The truncated IDN2 protein including both XS and coiled-coil domains was sufficient for binding to the two forms of double-stranded RNAs, whereas the zinc finger and XH domains were not required for the RNA-binding ability (Figure 6C). These results are different from those described in a previous study in which IDN2 could specifically bind 5′ overhanging double-stranded RNA but not the double-stranded RNA with blunt ends [45].

The bacterially purified IDP1 and IDP2 was incapable of binding double-stranded RNA and any other form of nucleic acids (Figure 6D). The binding of IDN2, IDP1, and IDP2 with double-stranded RNA was further tested when an increasing amount of each full-length protein was added to the RNA-binding reaction system. The results showed that IDN2 but not IDP1 and IDP2 was able to specifically bind both 5′ overhanging double-stranded RNA and blunt-end double-stranded RNA (Figure 6E), suggesting that IDP1 and IDP2 may have roles other than the nucleic acid-binding ability in the IDN2-IDP1/2 complex.

## Discussion

IDN2 has been previously identified by two independent forward genetic screens and has been demonstrated to be an important component that may function at a downstream step in the RdDM pathway [45], [46]. Our results suggest that IDN2 and its paralogs, IDP1 or IDP2 form a complex required for RdDM. The DDR complex is another important component that acts at a downstream step in the RdDM pathway [39]. Our results show, however, that IDN2 does not associate with DMS3, a component of the DDR complex, suggesting a different role for IDN2 at the downstream of RdDM. To further characterize the IDN2-IDP1/IDP2 complex, we generated *IDP1-3xFlag* transgenic plants and carried out affinity purification of IDP1-3xFlag. Mass-spectrometric analysis showed that the IDP1-3xFlag co-purified proteins included IDN2 as well as IDP2 (Figure S11 and Table S2), suggesting that IDP1 and IDP2 can exist in the same IDN2-containing complex. Furthermore, because the *idp2* single mutant has no clear DNA methylation defect compared to *idp1* (Figure 4 and Figure S4), we propose that that IDP1 and IDN2 may also form a functional complex without IDP2.

IDN2 is a member of a protein family that contains nine proteins. As indicated by phylogenetic analysis (Figure 1E), IDN2 and two other proteins, AT4G01780 and AT3G12550 are highly similar and classified into the same clade. The proteins AT1G15910 and AT4G00380 have low similarity with IDN2 and belong to another clade. In this study, we show that IDP1 (AT1G15910) and IDP2 (AT4G00380) interact with IDN2 and form a complex required for RdDM. IDN2 can form a homodimer by itself, but it is unable to associate with its most similar homologs AT4G01780 and AT3G12550 as indicated by affinity purification. Our result suggests that IDN2 interaction with IDP1 and IDP2 does not rely on the sequence similarity between IDN2 and IDP1 or IDP2. It follows that interaction with IDN2 evidently requires some sequence specificities that are present in IDP1 and IDP2 but are absent in the IDN2 homologs AT4G01780 and AT3G12550.

The coiled-coil domain has been demonstrated to be required for dimerization of many proteins [52]. Our yeast two-hybrid results show that IDN2 depends on its coiled-coil domain in order to form a homodimer, whereas the XH domain but not the coiled-coil domain is required for the association of IDN2 with its paralogs IDP1 and IDP2 (Figure 2B, 2C). The results reveal that IDN2 associates with itself and its paralogs through the coiled-coil domain and the XH domain, respectively. Both interaction domains seem to be required for the formation of a functional IDN2-IDP1/2 complex in RdDM *in vivo*. According to gel filtration, the IDN2 fusion protein-containing complex is ∼400 KDa, corresponding to a tetramer of IDN2 and its paralogs IDP1 and IDP2 (Figure 1D). We propose that in the IDN2-containing tetramer, two IDN2 proteins form a homodimer through their coiled-coil domains in parallel, whereas two IDN2 paralogs IDP1 and/or IDP2 separately associate with the two C-terminal XH domains of the IDN2 dimer. Because the yeast two-hybrid assay detected no interaction between the IDP1/2 proteins, the two IDP1/2 proteins in the tetramer are unlikely to directly interact with each other.

The IDN2 XH domain has been demonstrated to be sufficient for IDN2 interaction with IDP1/2 by yeast two-hybrid assay (Figure 2B, 2C). The IDN2E600Q mutation in XH domain reduced the IDN2-IDP1/2 interaction in yeast two-hybrid assay as well as in the IDN2-6xMyc pull-down assay (Figure 2D and Figure 3C), demonstrating the important role of the IDN2 XH domain in the IDN2-IDP1/2 interaction. However, the IDN2W616A mutation had no effect on the IDN2-IDP1/2 interaction in yeast two-hybrid, whereas it reduced the IDN2 association with IDP1/2 in the IDN2-6xMyc pull-down assay (Figure 2D and Figure 3C). The results suggest that the interaction between the IDN2 XH domain and the IDP1/2 proteins is insufficient for the formation of the IDN2-IDP1/IDP2 complex *in vivo*. It is possible that IDN2W616 in the XH domain is required for proper assembly of the IDN2-IDP1/IDP2 complex, whereas IDN2E600 is a site that directly interacts with IDP1 or IDP2.

Our EMSA result shows that IDN2 can bind both 5′ overhanging double-stranded RNA and blunt-end double-stranded RNA (Figure 6B, 6E), which is inconsistent with a previous report that IDN2 can bind 5′ overhanging double-stranded RNA but not any other types of nucleic acids [45]. The inconsistent results may be due to the different double-stranded RNA probes used in EMSA. The previous study used blunt-end double-stranded RNA with 21-base pairs [45], which may be too short for IDN2 binding. Alternatively, the double-stranded RNA-binding ability of IDN2 may depend on RNA-sequence specificities. Our results show that both the full-length IDN2 and the truncated IDN2 containing the XS and coiled-coil domains are capable of binding two types of double-stranded RNAs (Figure 6A–6C), suggesting that the 5′ overhangs of double-stranded RNA are not required for IDN2 binding by EMSA. However, the RNA substrates that are bound by IDN2 *in vivo* require further investigation.

Unlike IDN2, the IDN2 paralogs IDP1 and IDP2 had no double-stranded RNA binding ability in our EMSA experiment (Figure 6D, 6E), although the sequences of IDP1 and IDP2 are similar to those of IDN2. We propose that in the IDN2-IDP1/2 complex, IDN2 is responsible for binding double-stranded RNA substrates, whereas its paralogs IDP1 and IDP2 play different roles. Our results show that both IDP1 and IDP2 are required for DNA methylation (Figure 4, Figure S4 and Figure S9), suggesting that the IDN2-IDP1/2 complex is required for RdDM *in vivo*. IDN2 and IDP1 or IDP2 cooperate in the IDN2-IDP1/2 complex and regulate the RdDM pathway together. Although further research is required to explore the exact function of IDN2 and IDP1/2 *in vivo*, this study has clearly demonstrated that the newly characterized IDN2-IDP1/2 complex is essential for RdDM.

During this manuscript was under review, Xie et al. reported that two IDN2-like proteins, FACTOR OF DNA METHYLATION 1 (FDM1) and FDM2 are functionally redundant with IDN2 in RdDM [55]. FDM1 and FDM2 are synonymous with IDP1 and IDP2, respectively. The identification of the same two proteins, IDP1/FDM1 and IDP2/FDM2 as the most important IDN2 homologs in RdDM by two independent studies further underlies the importance of IDP1/FDM1 and IDP2/FDM2 in the RdDM pathway. More importantly, in our study, we characterized an IDN2-IDP1/IDP2 complex required for the RdDM pathway. IDP1 and IDP2 belong to a different subfamily from IDN2 and its close homologs (AT3G12550 and AT4G01780) (Figure 1E). IDN2 interacts with itself via the coiled-coil domain, whereas it interacts with IDP1 and IDP2 via the XH domain (Figure 2B–2D). Moreover, unlike IDN2, IDP1 and IDP2 are incapable of binding double-stranded RNA (Figure 6D, 6E). These results suggest that IDN2 and IDP1 or IDP2 have different biochemical functions in the IDN2-IDP1/IDP2 complex.

In the case that the IDN2 close homolog, AT1G12550 (FDM3) acts a role in RdDM [55], FDM3 is likely to have redundant biochemical functions with IDN2. Disruption of the IDN2-IDP1/IDP2 complex by the *idn2* mutation may be partially rescued by the potential FDM3-IDP1/IDP2 complex. However, when both IDN2 and IDP1 or IDP2 were knocked out in the double mutants, *idn2idp1* and *idn2idp2*, the function of the IDN2-IDP1/IDP2 complex was undoubtedly much more difficult to be rescued by their homologs. Therefore, it is reasonable that the double mutants, *idn2idp1*/*idn2fdm1* and *idn2idp2*/*idn2fdm2* cause strong reduction in DNA methylation and siRNA accumulation than each of single mutants as indicated by Xie et al. [55]. Taken together, our biochemical results are consistent with the reverse genetic analysis results reported by Xie et al. [55] and provide further insights into the functioning mechanism of IDN2 and its paralogs IDP1 and IDP2.

## Materials and Methods

### Plant materials, constructs, and *Arabidopsis* transgenic lines

*Arabidopsis* plant materials were grown on MS plates under long-day conditions at 22°C. When adult plants or flowers were needed, the plants were transplanted from MS plates into soil and grown in a growth room under long-day conditions. The plants used in this study included the wild-type Col-0, *nrpd1-3* (SALK_128428), *idn2-5/rdm12-2* (FLAG_550B05), *idp1-1* (SALK_075378), *idp2-1* (SALK_066712), *idp1-1idp2-1*, and the wild-type C24, *ros1-1*, and *ros1-1rdm12-1/ros1-1idn2-4* that contain an *RD29A* promoter-driven luciferase reporter transgene.

The full-length *IDN2* genomic sequence was amplified and cloned into the binary pCAMBIA1300 vector with its C-terminal tagged by *6xMyc* (Figure S12A, S12C). The full-length *IDP1* genomic sequence was cloned into the vector modified from pCAMBIA1305, in which the 35S promoter-driven GUS reporter gene was replaced by *3xFlag* (Figure S12B, S12D). Site-directed mutagenesis was carried out to generate the mutated *IDN2* sequences. The mutated *IDN2* sequence was cloned into the same vector with the wild-type *IDN2* sequence. The constructs were transformed into wild type and *ros1idn2-4* by agrobacteria infection. The T1 transgenic plants were grown on MS medium supplemented with 20 µg/ml hygromycin, and the resistant positive seedlings were grown for further analysis.

### Affinity purification, mass spectrometry, and gel filtration

Three grams of flowers or seedlings from *IDN2-Myc* or *IDP1-3xFlag* transgenic plants as well as from wild type were harvested and ground in liquid nitrogen. The ground materials were homogenized in 15 ml of lysis buffer (50 mM Tris [pH 7.6], 150 mM NaCl, 5 mM MgCl_2_, 10% glycerol, 0.1% NP-40, 0.5 mM DTT, 1 mM PMSF, and 1 protease inhibitor cocktail tablet/50 ml [Roche]). Following centrifugation, each supernatant was incubated with 100 µl of Anti-c-Myc Agarose (Sigma, A 7470) or Anti-Flag M1 Agarose (Sigma, A 4596) at 4°C for 2.5 h. The resins were washed twice for 5 min with 10 ml of lysis buffer and then five times for 5 min with 1 ml of lysis buffer. The Myc beads-bound proteins were eluted with 0.1 M ammonium hydroxide at pH 11.5, whereas the Flag beads-bound proteins were eluted with 3xFlag peptide (Sigma, F 4799).

The eluted fraction was run on a 12% SDS-PAGE gel, followed by silver staining with the ProteoSilver Silver Stain Kit (Sigma, PROT-SIL1). Either whole proteins or individual bands were cut from the gel and purified. The purified peptides were eluted on an analytical capillary column (50 µm×10 cm) packed with 5-µm spherical C18 reversed-phase material (YMC, Kyoyo, Japan). The eluted peptides were sprayed into a LTQ mass spectrometer (Thermo Fisher Scientific) equipped with a nano-ESI ion source. Database searches were performed on the Mascot server (Matrix Science Ltd., London, UK) against the IPI (International Protein Index) *Arabidopsis* protein database. Mapped peptides were calculated and shown.

For gel filtration, 0.4 g of plant material was ground in liquid nitrogen and suspended in 2.4 ml of lysis buffer and centrifuged at 13200 rpm for 10 min at 4°C. The supernatants were then passed through a 0.22-µm filter, and 250 µl was loaded onto a Superose 6 10/300 GL column (GE Healthcare, 17-5172-01); 500-µl fractions were collected. A 10-µl volume of every fraction was run on 10% SDS-PAGE for western blot assay of IDN2-Myc and DMS3. The column was calibrated with the standard proteins.

### Yeast two-hybrid assay

For yeast two-hybrid analysis, the full-length cDNAs of *IDN2* and its paralogs *IDP1* and *IDP2* were cloned into pGADT7 and pGBKT7 vectors, respectively. The yeast strain PJ694a was co-transformed with corresponding pGADT7 and pGBKT7 constructs, and grown on the synthetic dropout medium SD-trp-leu for selection. The positive strains were incubated in SD-trp-leu liquid medium at 28°C, and serial decimal dilutions were used for spot assay on SD-trp-leu-his plates supplemented with 5 mM to 20 mM 3-AT. The addition of 3-AT to the medium increases the stringency of selection. The interaction between GAL4-AD fusion proteins and GAL4-BD fusion proteins activates the expression of the reporter gene HIS, which promotes the growth of the strain on SD-trp-leu-his plates supplemented with 3-AT. To identify the IDN2 domain that is required for dimerization, we cloned the truncated and mutated *IDN2* cDNAs into the pGBKT7 vector and co-transformed them with pGADT7-*IDN2*, pGADT7-*IDP1*, or pGADT7-*IDP2*. The positive strains were incubated in SD-trp-leu liquid medium and were used for spot assay on SD-trp-leu-his medium supplemented with 3-AT.

### DNA methylation assay

Chop-PCR, southern blotting, and bisulfite sequencing were conducted to test DNA methylation. For chop-PCR, genomic DNA was digested with the DNA methylation-sensitive restriction enzyme HaeIII, followed by amplification of target DNA sequences. For southern blotting, genomic DNA was digested with HaeIII, and 20 µg of the digested DNA was run on a 1.0% agarose gel at 40 V overnight followed by southern blotting. For bisulfite sequencing, 2 µg of genomic DNA was treated with sodium bisulfite, which converts unmethylated cytosines to uracils. The converted genomic DNA was purified with the EpiTect Bisulfite Kit (Qiagene), followed by amplification and cloning. The cloned sequences were analyzed online by CyMATE (http://www.gmi.oeaw.ac.at/research-groups/cymate) [56]. At least 15 individual clones were collected for sequencing for each sample. A summary of the bisulfite sequencing results is shown in Table S3. The primers used for DNA methylation assay are described in Table S4.

### Detection of RNA transcripts and small RNAs

For semiquantitative RT-PCR, RNA was isolated from 2-week-old seedlings on MS plates as described previously [57]. The isolated RNA was treated with DNase to remove DNA contamination and used for RT-PCR. For RT-PCR of transposable elements, sequence-specific primers were used as reverse primers to generate the first-stranded cDNAs. For RT-PCR of protein-encoding genes, oligo-dT was used as a reverse primer. The constitutively expressed gene ACT7 or *TUB4* was amplified as internal controls. Amplification of RNA samples without reverse transcription (No RT) was carried out to test whether the RNA samples were contaminated with DNA. For small RNA blotting, small RNAs were extracted as described previously [57] and run on a 15% polyacrylamide gel at 200 V for 3–4 h. The small RNA gel was ethidium bromide-stained for imaging and electrotransferred to Hybond-N+ membranes (Amersham) for small RNA hybridization. Small RNA probes were [γ-32P]ATP-labeled DNA oligonucleotides or [α-32P]dCTP-labeled PCR products. Small RNA hybridization was carried out in PerfectHyb buffer (Sigma) overnight at 38°C. The DNA oligonucleotides that were used are described in Table S4.

### Electrophoretic mobility shift assay

The following were cloned into the pET28a or pET30 vector: full-length *IDN2* cDNA; truncated *IDN2* cDNAs *IDN2-a*, *IDN2-b*, and *IDN2-c*; and full-length *IDP1* and *IDP2* cDNAs. Each construct was transformed into *E. coli* BL21 for expression of His fusion proteins. His fusion proteins were purified by Ni-NTA His Bind Resin (Novagen) and used for electrophoretic mobility shift assay (EMSA). EMSA assays were performed as previously described [33]. For the binding assay, RNA and DNA oligonucleotides were synthesized and end labeled with T4 polynucleotide kinase and [γ-32P]ATP. The labeled oligonucleotides were purified with G-25 columns (GE Healthcare) and used as probes in the binding assay. The binding reaction included 4 µl of labeled RNA or DNA probes, 2 µg of His fusion protein, 25 mM HEPES (pH 7.6), 50 mM KCl, 0.1 mM EDTA (pH 8.0), 12.5 mM MgCl_2_, 1 mM DTT, 0.5% (w/v) BSA, and 5% (w/v) glycerol. The reaction mixtures were incubated at room temperature for 30 min and run on 4% nondenaturing polyacrylamide gels at 200 V for 2 h. Gels were exposed to X-ray film for analysis.

## Supporting Information

Figure S1 Sequence alignment of IDN2 and eight IDN2-like proteins in the IDN2 family in *Arabidopsis*.
(PDF)

Figure S2 Sequence alignment of IDN2 and the two IDN2-interacting paralogs IDP1 and IDP2.
(TIF)

Figure S3 Genotyping and confirmation of the *idp1* and *idp2* mutants. (A) Diagram of *IDP1* and *IDP2* genes and their mutants. The T-DNA insertions in *idp1-1* (Salk_075378) and *idp2-1* (Salk_066712) are shown. Exons (boxes), introns (line), and open reading frame (solid boxes) are indicated. (B) Genotyping of the *idp1-1* and *idp1-2* mutants. The gene-specific primers flanking T-DNAs were used for amplification of *IDP1* and *IDP2*. No amplification indicates that the materials are homozygous. (C) The RNA transcript levels of *IDP1* and *IDP2* were detected by semiquantitative RT-PCR in the *idp1-1* and *idp2-1* mutants. Amplification of *TUB4* was used as an internal control.
(TIF)

Figure S4 Effect of *idp1* and *idp2* on DNA methylation. (A) Effect of *idp1* and *idp2* on the DNA methylation level of *AtSN1* and *IGN5* was determined by chop-PCR. Genomic DNA was digested by the DNA methylation-sensitive restriction enzyme HaeIII, followed by amplification. (B) Effect of *idp1* and *idp2* on *AtMU1* DNA methylation was tested by southern blotting. Genomic DNA was digested by HaeIII followed by southern hybridization.
(TIF)

Figure S5 Diagram of the bisulfite sequencing results of *AtSN1*. The bisulfite sequencing results were analyzed by CyMATE. (A) The positions of all cytosines in the tested *AtSN1* sequence. (B) The methylation status of all cytosines for each clone in WT, *nrpd1*, *idn2-5*, *idp1*, and *idp2* is shown. The cytosine methylation in different contexts (CG, CHG, and CHH) is diagramed as indicated. Each line represents the cytosine methylation status for each clone. Class 1, Class 2, and Class 3 represent the cytosines at CG, CHG, and CHH sites, respectively. “H” is A, T, or C.
(TIF)

Figure S6 Diagram of the bisulfite sequencing results of *MEA-ISR*.
(TIF)

Figure S7 Diagram of the bisulfite sequencing results of *Solo LTR*.
(TIF)

Figure S8 The *IDP1-3xFlag* transgene is functional *in vivo*. (A) The expression of the *IDP1-3xFlag* transgene in wild type and *idp1* was tested by the Flag antibody. Ponceau S staining of Rubisco is shown as a loading control. (B) The *IDP1-3xFlag* transgene complements the DNA methylation defect of *AtSN1*. *AtSN1* methylation was tested by chop-PCR.
(TIF)

Figure S9 Detection of DNA methylation in the *idp1idp2* double mutant. The DNA methylation level of *AtSN1* and *IGN5* was determined by chop-PCR. Genomic DNA from each indicated genotype was digested by the DNA methylation-sensitive restriction enzyme HaeIII, followed by amplification.
(TIF)

Figure S10 Each labeled nucleic acid was run on native gel when no protein was added. The result indicated that all nucleic acids were well labeled and purified. The identifications of probes (1–6) were shown in Figure 6B.
(TIF)

Figure S11 Co-purified proteins by IDP1-3xFlag pull down. Protein extracts were isolated from the *IDP1-3xFlag* transgenic plants as well as from the wild-type control. The extracts were subjected to affinity purification of IDP1-3xFlag by the Flag antibody. Total purified proteins were visualized by silver staining on SDS-PAGE gel.
(TIF)

Figure S12 Diagram of the *IDN2-6xMyc* and *IDP1-3xFlag* constructs. (A, B) The backbones of the *IDN2-6xMyc* and *IDP1-3xFlag* constructs are pCAMBIA1300 and pCAMBIA1305, respectively. (C) Diagram of the *IDN2-6xMyc* construct. A 6xMyc encoding DNA sequence and a NOS terminator were inserted between XbaI and HindIII restriction sites. The native promoter-driven *IDN2* genomic sequence was cloned in frame with the 6xMyc. (D) Diagram of the *IDP1-3xFlag* construct. The 35S promoter-driven *GUS* reporter was replaced by a 3xFlag encoding sequence between HindIII and BstEII restriction sites. The native promoter-driven *IDP1* genomic sequence was cloned in frame with the *3xFlag* between EcoRI and SalI sites.
(TIF)

Table S1 List of all identified peptides from mass-spectrometric analysis. All listed peptides are significant matches (p<0.05) and considered unambiguous. 1) Numbers of matches for each peptide. 2) Unique peptides are labelled with “U”. Numbers of total and unique peptide matches for corresponding proteins are shown in parentheses.
(XLS)

Table S2 Mass-spectrometric analysis of IDP1-3xFlag affinity purification. Total protein extracts were isolated from the IDP1-3xFlag transgenic plants in the wild-type background and subjected to affinity purification of IDP1-3xFlag. The co-purified proteins were purified and used for mass-spectrometric analysis. Mascot score of each protein is shown. 1) The number is the total number of mass spectra matched to the corresponding protein, including the redundant ones that match to the same peptides. 2) The number is deduced from the “Matched queries” by removing the redundant peptides. 3) The number is calculated from “Matched queries” by removing the overlapped peptide sequences among its homologous protein family.
(DOC)

Table S3 Summary of bisulfite sequencing results. The bisulfite sequencing results for *AtSN1*, *MEA-ISR*, and *Solo LTR* is summarized. The number of analyzed clones for each genotype and the number of each type of cytosine contexts are shown. The percentage of methylated cytosines for each cytosine context is indicated for each genotype. The numbers of methylated cytosines and total cytosines for each type of cytosine contexts are shown as “methylated cytosines/total cytosines” in parentheses.
(DOC)

Table S4 List of DNA and RNA oligonucleotides used in this study.
(XLS)
