# Supplementary material for: IDN2 and Its Paralogs Form a Complex Required for RNA–Directed DNA Methylation
Source: PLoS Genet. 2012 May 3;8(5):e1002693. doi: 10.1371/journal.pgen.1002693 (PMC3342958; doi:10.1371/journal.pgen.1002693)
Supplement: Figure S1 — Sequence alignment of IDN2 and eight IDN2-like proteins in the IDN2 family in Arabidopsis. (PDF) [file pgen.1002693.s001.pdf]

```
At3g48670  1    MGSTVILSSDDEDSDISESEMDEYGDKMYLNLKGGKLKVRLSPQAFICPYCPNKKKTSFQYKDLLQHASG
At3g12550  1    MN--NKLSD--------------FEKNLYKKLKSGKLEVKVSYRTFLCPYCPDNKKKVGLYVDILQHASG
At4g01780  1    MGGTRHCYG--------------LGYEMFN-------FVWSSSVRFVWSSS----ETLFKIVIITRSLCI
At1g15910  1    MSIS-DEEAE-----ISESEIEDYSETPYRLLRDGT-YKVKVNGQLRCPFCAGKKKQDYKYKELYAHATG
At4g00380  1    MDIS-DEESE-----ISESEIEEYSKTPYHLLRSETYYKVKVNGRLRCPFCVGKKKQDYKYKELHAHATG
At1g13790  1    MYSRRELEDL--------------EYRYYSEMKDGTRKVKISESLFRCPFCYIDRKRDYQFDDLLRHASG
At1g80790  1    MDNSSDEESE-----ISESEIDVYYEKPYEKLMNGD-YKVKVKDTFRCPFCAGKKKQHYKYKELLAHASG
At5g59390  1    ----------------------------------------------------------------------
At4g01180  1    ----------------------------------------------------------------------

At3g48670  71   VGNSNSDKR-------------------------------------------------------------
At3g12550  55   VGNSQSKKR-------------------------------------------------------------
At4g01780  46   LGKVHT----------------------------------------------------------------
At1g15910  64   VSKGSATR--------------------------------------------------------------
At4g00380  65   VSKGSATR--------------------------------------------------------------
At1g13790  57   IGGSSRTKDGRDKARHLALERYMRKYLRPRERPRPSPTSDVSSLPKEEFTGKWKSTLSTTEEGEFITTEN
At1g80790  65   VAKGSASR--------------------------------------------------------------
At5g59390  1    ----------------------------------------------------------------------
At4g01180  1    ----------------------------------------------------------------------

At3g48670  79   ---------------------------SAKEKASHLALVKYLQQDLADSASEAEPSSKRQKN--------
At3g12550  63   ---------------------------SLTEKASHRALAKYLIKDLAHYATSTISKRLKARTS-------
At4g01780  51   ---------------------------LLTFKVLTFLLKIELQCVFSDCVYYVAET--------------
At1g15910  71   ---------------------------SALQKANHLALAMFLENELAGYAEPVPRPPVVPPQ--------
At4g00380  72   ---------------------------SALQKSNHLALAKFLENDLAGYAEPLPRPPVVPPL--------
At1g13790  127  SSSPHIVKAEPKFVSGDDSGRSGEERLKFSDKPDPFFSNEDKSYPAKRPCLVSGAKEGDEPVQRIGLSHG
At1g80790  72   ---------------------------SAKQKANHFALAKYMENELAGDAD-VPRPQIPSSS--------
At5g59390  1    --------------------------MEKNQQKQEFLAETSEMKNTKNNDDCSVQ---------------
At4g01180  1    --------------------------MKKKQQKQEFVAETSEMN---NNNECSGQD--------------

At3g48670  114  ----------GNPIQDCDHDEKLVYPWKGIVVNIPTTKAQDGRS-AGESGSKLRDEYILRGFNPTRVRPL
At3g12550  99   --FIPAETGDAPIIYDDAQFEKLVWPWKGVLVNIPTTSTEDGRSCTGESGPKLKDELIRRGFNPIRVRTV
At4g01780  79   ----------------------------GIVVNIPTTKAQDGRP-AGESGSKLRDEYILRGLN-------
At1g15910  106  -----------LDETEPNPHNVYVWPWMGIVVNP--LKEADDKELLLDSAYWLQ---TLSKFKPIEVNAF
At4g00380  107  -----------LDETEPNPHNVYVWPWMGIVVNP--LKETDDKELLLDSVYWLQ---TLSKFKPVEVNAF
At1g13790  197  ASFAPTYPQKLVSLGAGNGDQMYVHPWKGILANMKRTFNEKTRKYAGESGSKIREDLIKKGFNPHKVTPL
At1g80790  106  -----------TEQSQAVVDDIYVWPWMGIVINP--VRRTDNKNVLLDSAYWLK---KLARFNPLEVKTL
At5g59390  29   ------------------QQQRFVWPWVGLVANIP-TEVEPSGRRVGKSGSTLRDEFTMKGFNPTRVQPI
At4g01180  27   ------------------QQKRYVWPWVGLVANVP-TEVEPSGRRVGKSGSTLRDEFTLKGFNPTRVKPI

At3g48670  174  WN-YLGHSGTAIVEFNKDWNGLHNGLLFDKAYTVDGHGKKDWLKKDGPKLG-LYGWIARADDYNGNNIIG
At3g12550  168  WD-RFGHSGTGIVEFNRDWNGLQDALVFKKAYEGDGHGKKDWLCG-ATDSS-LYAWLANADDYYRANILG
At4g01780  113  -----------------------NGLLFDKAYRVDGHGKKDWMKKDGPKLG-LYGWIAGADDYNVDGREG
At1g15910  161  WV-EQDSIVGVIAKFNGDWSGFAGATELEKEFETQGSSKKEWTERSGDSESKAYGWCARADDFESQGPIG
At4g00380  162  WV-EQDSIVGVIAKFDSDWSGFAAATELEKEFETQGSCKKEWTERSGDSESKAYGWCARADDFQSQGPIG
At1g13790  267  WNGRLGFTGFAIVDFGKEWEGFRNATMFDKHFEVSQCGKRDHDLTRDPGD-KLYGWVAKQDDYYSRTAIG
At1g80790  161  WL-DQESVVAVIPQFNSGWSGFKSVTELEKEYEIRGCGRKDWIDKRGDWRSKAYGWCARADDYNSQGSIA
At5g59390  81   WD-FKGHSGFALVEFAKDFEGFESAMNFERSFNLDRHGKRDWEKGHRLRDNKLYGWVAREDDYNRSDTVG
At4g01180  79   WN-TKGHTGFALVEFAKDFKGFESAMQFEKSFDLDRHGKRDWKKGHRLRDDKLYGWLAREDDYNRSDTVG

At3g48670  242  ENLRKTGDLKTIAELTEEEARKQELLVQNLRQLVEEKKKDMKEIEELCSVKSEELNQLMEEKEKNQQKHY
At3g12550  235  ENLRKMGDLKSIYRFAEEEARKDQKLLQRLNFMVENKQYRLKKLQIKYSQDSVKLKYETEEKEKILRAYS
At4g01780  160  E----------------ESAK-------------------------------------------------
At1g15910  230  EYLSKEGQLRTVSDISQKNVQDRNTVLEELSDMIAMTNEDLNKVQYSYNRTAMSLQRVLDEKKNLHQAFA
At4g00380  231  EYLSKEGTLRTVSDILQNNVQDRNTLLDVLSNMIDMTNEDLNKAQHSYNRTAMSLQRVLDEKKNLHQAFA
At1g13790  336  DHLRKQGDLKSVSGKEAEDQRKTFTLVSNLENTLVTKSDNLQQMESIYKQTSSVLEKRMKEKDEMINTHN
At1g80790  230  EYLSKVGKLRSFSDITKEEIQNKSIVVDDLANKIAMTNEDLNKLQYMNNEKTLSLRRVLIEKDELDRVYK
At5g59390  150  KNVKKKRDLKSISQIVEEDERKMVHLVENMSQTIEKKKQSKQELEQKVDETSRFLESLELHNVLLNKNYQ
At4g01180  148  KNVKKKRDLKSISQIVEEDQRKLYHLFENMCQTIEKNKQRKQQLEQKVDET---LESLEFHNLMLNNSYQ
```

```
At3g48670  312 RELNAIQERTMSHIQKIVDDHEKLKRLLESERKKLEIKCNELAKREVHNGTERMKLSEDLEQNASKNSSL
At3g12550  305 EDLTGRQQKSTDHFNRIFADHEKQKVQLESQIKELEIRKLELAKREAENETQRKIVAKELEQNAAINSYV
At4g01780  164 --LNAIQERTMRHIQKIVDDHERLTKLLESEKKKLEIKGNELAKPQVHNGTERMKLSEDLEQ--------
At1g15910  300 DETKKMQQMSLRHIQKILYDKEKLSNELDRKMRDLESRAKQLEKHEALTELDRQKLDEDKRKSDAMNKSL
At4g00380  301 EETKKMQQMSLRHIQRILYDKEKLRNELDRKMRDLESRAKQLEKHEALTELERQKLDEDKRKSDAMNKSL
At1g13790  406 EKMSIMQQTARDYLASIYEEHEKASQHLEAQRKEYEDRENYLDKCQAKNKTERRKLQWQKHKNLMATQEQ
At1g80790  300 QETKKMQELSREKINRIFREKERLTNELEAKMNNLKIWSKQLDKKQALTELERQKLDEDKKKSDVMNSSL
At5g59390  220 EGFQKMQMKMEELYQQVLDGHEKSLAELEAKREKLDERARLIEQRAIINEEEMEKSRLEREMNQKAMCEQ
At4g01180  215 EEIQKMEKNMQEFYQQVLGGHEKSFAELEAKREKLDERARLIEQRAIKNEEEMEKTRLEREMIQKAMCEQ

At3g48670  382 ELAAMEQQKADEEVKKLAEDQRRQKEELHEKIIRLERQRDQKQAIELEVEQLKGQLNVMKHMA--SDGDA
At3g12550  375 QLSALEQQKTREKAQR---LAVDHKEKLHKRIAALERQLDQKQELELEVQQLKSQLSVMRLVE--LDSGS
At4g01780  224 ---------------------RQKEELHEKIIRLERQIDQVQAIELEVEQLKGQLNVMKHMA--SDGDA
At1g15910  370 QLASREQKKADESVLRLVEEHQRQKEDALNKILLLEKQLDTKQTLEMEIQELKGKLQVMKHLG--DDDDE
At4g00380  371 QLASREQKKADESVLRLVEEHQRQKEDALNKILLLEKQLDTKQTLEMEIQELKGKLQVMKHLG--DDDDE
At1g13790  476 NKADEDMMRLAEQQQ-------REKDELRKQVRELEEKIDAEQALELEIERMRGDLQVMGHMQEGEGEDS
At1g80790  370 QLASLEQKKTDDRVLRLVDEHKRKKEETLNKILQLEKELDSKQKLQMEIQELKGKLKVMKHE---DEDDE
At5g59390  290 NEANEEAMKLAEKHQA--SSSLKEKEKLHKRIMEMEAKLNETQELELEIEKLKGTTNVMKHMVGSDG-DK
At4g01180  285 NEANEEAMKLAEKHQ-------KEKEKLHKRIMEMEAKLNETQELELEIEKLKGTTNVMKHMVGCDG-DK

At3g48670  450 EVVKEVDIIFKDLGEKEAQLADLDKFNQTLILRERRTNDELQEAHKELVNIMK--EWN---TNIGVKRMG
At3g12550  440 EIVNKVETFLRDLSETEGELAHLNQFNQDLVVQERKSNDELQEARRALISNLR--DMG---LHIGVKRMG
At4g01780  271 QVVKEVDIIFKDLVEKEAELADLNKFNQTLILRERRTNDELQEARKELVN-----------------CMG
At1g15910  438 AVQKKMKEMNDELDDKKAELEGLESMNSVLMTKERQSNDEIQAARKKLIAGLT--GLLGAETDIGVKRMG
At4g00380  439 AVQTKMKEMNDELDDKKAELEDLESMNSVLMTKERQSNDEIQAARQKMIAGLT--GLLGAESDIGVKRMG
At1g13790  539 KIKEMIEKTKEELKEKEEDWEYQESLYQTLVVKHGYTNDELQDARKALIRSMR---ELTTRAYIGVKRMG
At1g80790  437 GIKKMKKMKEELEEKCSELQDLEDTNSALMVKERKSNDEIVEARKFLITELR--ELVSDRNIIRVKRMG
At5g59390  357 DIVEKIAKTQIQLDAQET---ALHEKMMTLARKERATNDEYQDVLKEMIQVWNANEELMKQEKIRVKRMG
At4g01180  347 DIVEKIAKTQIELDARET---ALHEKMMTLARKERATNDEYQDARKEMIKVWKANEELMKQEKIRVKIMG

At3g48670  515 ELVTKPFVDAMQQKYCQQ--DVEDRAVEVLQLWEHYLKDSDWHPFKRVKLENEDREV------------E
At3g12550  505 ELDTKPFMKAMRIKYCQE--DLEDWAVEVIQLWEEYLKDPDWHPFKRIKLETAETIV------------E
At4g01780  324 ELVRKPFVDAMQQKYCQE--DVEDRAVEVLQLWEHYINDPDWHPYKRVKLENQDREV------------E
At1g15910  506 ELDEKPFLDVCKLRYSAN--EAAVEAATLCSTWQENLKNPSWQPFK--HEGTGDGAE------------E
At4g00380  507 ELDEKPFLDVCKLRYSAN--EARVEAATLCSTWKENLKNPSWQPFK--REGTGDGAE------------E
At1g13790  606 ALDETPFKKVAKEKYP--AVEADKKAEELCSLWEEHLGDSAWHPIKVVEKDGIAK--------------E
At1g80790  505 ELEEKPFMTACRQRCTVEE-EAQVQYAMLCSKWQEKVKDSAWQPFK--HVGTGDRKK------------E
At5g59390  424 QLNPAPFLPAVMKKHKVTQSKAENKAMKLCSVWEANIGDVQWTPFRVDESDGTPK--------------R
At4g01180  414 ELNPAPFLPAVMNKH---------KAMMLCSVWAAEIGDVQWTPFRVDESDGTPKQKLHISQHSKCEMQR

At3g48670  571 VIDDRDEKLRELKADLGDGPYNAVTKALLEINEYNPSGRYITTELWNFKADKKATLEEGVTCLLDQWEKA
At3g12550  561 VIDEDDEKLRTLKNELGDDAYQAVANALLEINEYNPSGRYISSELWNFREDRKATLEEGVNSLLEQWNQA
At4g01780  380 VIDDRDEKLRELKADLGDGPYNAVTKALLEINEYNPSGRYITTELWNFKEDKRATLEEGVTCLLDQWEKA
At1g15910  560 VVDEDDEQLKKLKREWGKEVHNAVKTALVEMNEYNASGRYTTPELWNFKEGRKATLKEVITFISNDIKIL
At4g00380  561 VVDEDDEQLKKLKREWGKEVHNAVKAALVEMNEYNASGRYPTSELWNFKEGRKATLKEVITFISTDIKNL
At1g13790  660 ELNEEDEKLQELRKELGEEVYAAVTQALKERNEYNGSGRYIVPELWNFKQNRKASIKEGVVYLVNSWKQK
At1g80790  560 VVDEEDEEIKKLREEWGEEVKNAVKTALEELNEFNPSGRYSVPELWNSKQGRKATLKEVIDYITQQVKTL
At5g59390  480 VVDENDEKLRTLKNQYGEEVYNEVVRTKLEIEEHNASGSYVILELWNYEENRKATMEEATDVMLKIRSKL
At4g01180  475 VVDENDEKLRMLKNQYGEEVYSEVVRAKLEMEEHNASGSYETEELWNYEENRKATIEEITDVMLKIRSKL

At3g48670  641 KRKRGMA-----
At3g12550  631 KHLKS-------
At4g01780  450 KRKRGMA-----
At1g15910  630 KRKRT-------
At4g00380  631 KRKRT-------
At1g13790  730 KPKPKRR-----
At1g80790  630 KRRRA-------
At5g59390  550 AAMKNKRKRLEV
At4g01180  545 AAMKNKRKRP--
```
