# Supplementary figures and images for: IDN2 and Its Paralogs Form a Complex Required for RNA–Directed DNA Methylation
Source: PLoS Genet. 2012 May 3;8(5):e1002693. doi: 10.1371/journal.pgen.1002693 (PMC3342958; doi:10.1371/journal.pgen.1002693)

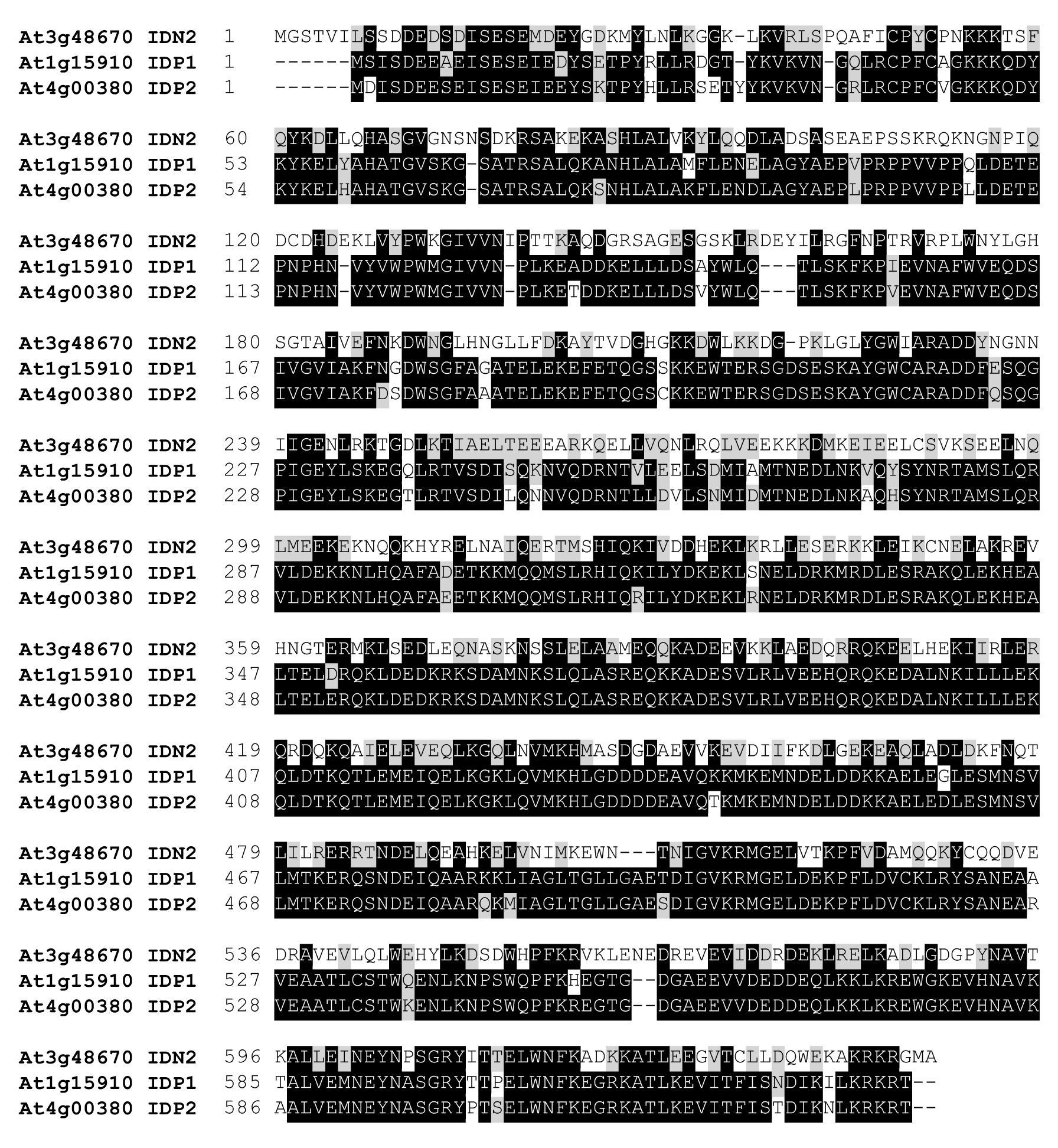

Supplement: Figure S2 — Sequence alignment of IDN2 and the two IDN2-interacting paralogs IDP1 and IDP2. (TIF) [file pgen.1002693.s002.tif]

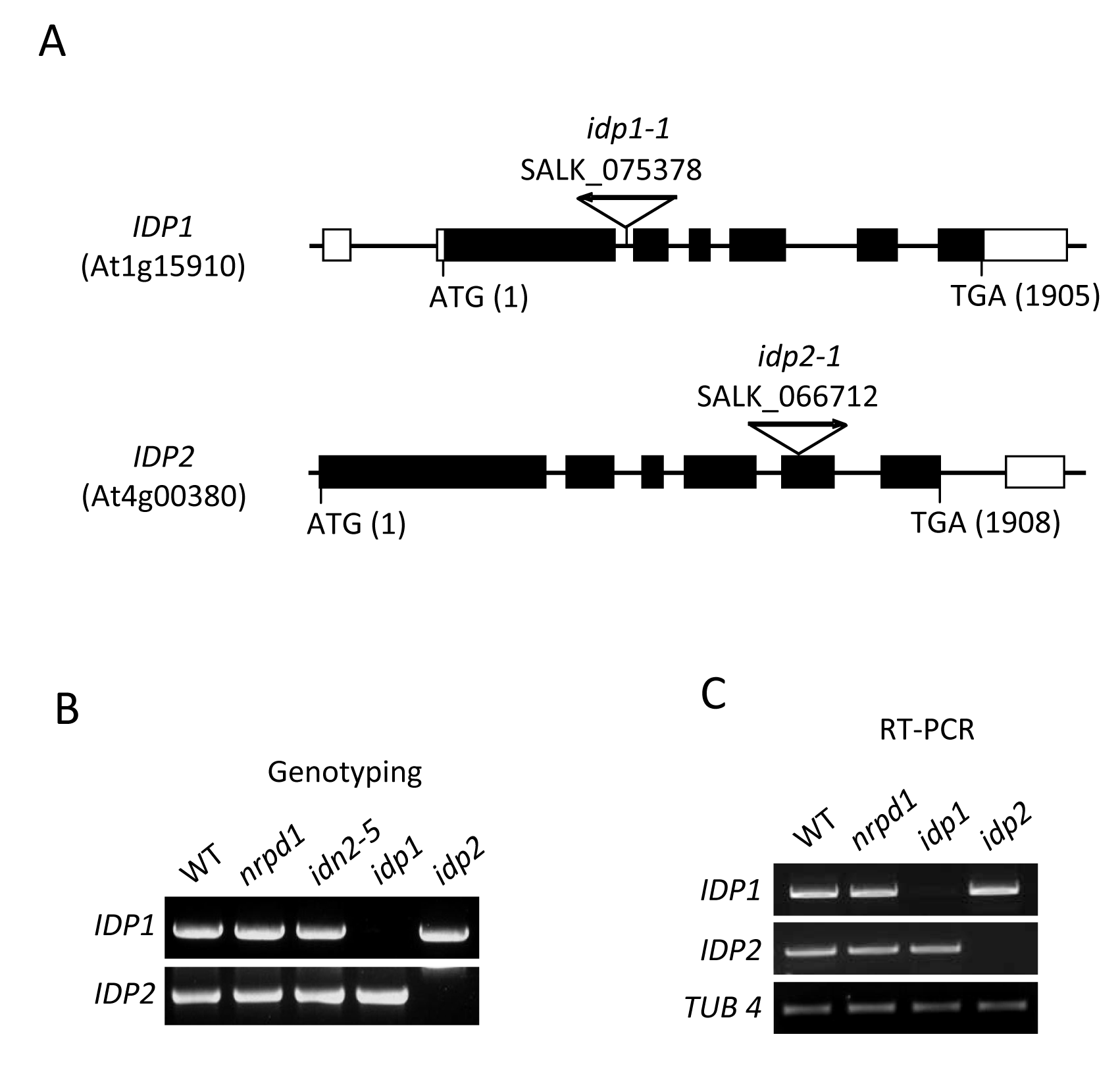

Supplement: Figure S3 — Genotyping and confirmation of the idp1 and idp2 mutants. (A) Diagram of IDP1 and IDP2 genes and their mutants. The T-DNA insertions in idp1-1 (Salk_075378) and idp2-1 (Salk_066712) are shown. Exons (boxes), introns (line), and open reading frame (solid boxes) are indicated. (B) Genotyping of the idp1-1 and idp1-2 mutants. The gene-specific primers flanking T-DNAs were used for amplification of IDP1 and IDP2. No amplification indicates that the materials are homozygous. (C) The RNA transcript levels of IDP1 and IDP2 were detected by semiquantitative RT-PCR in the idp1-1 and idp2-1 mutants. Amplification of TUB4 was used as an internal control. (TIF) [file pgen.1002693.s003.tif]

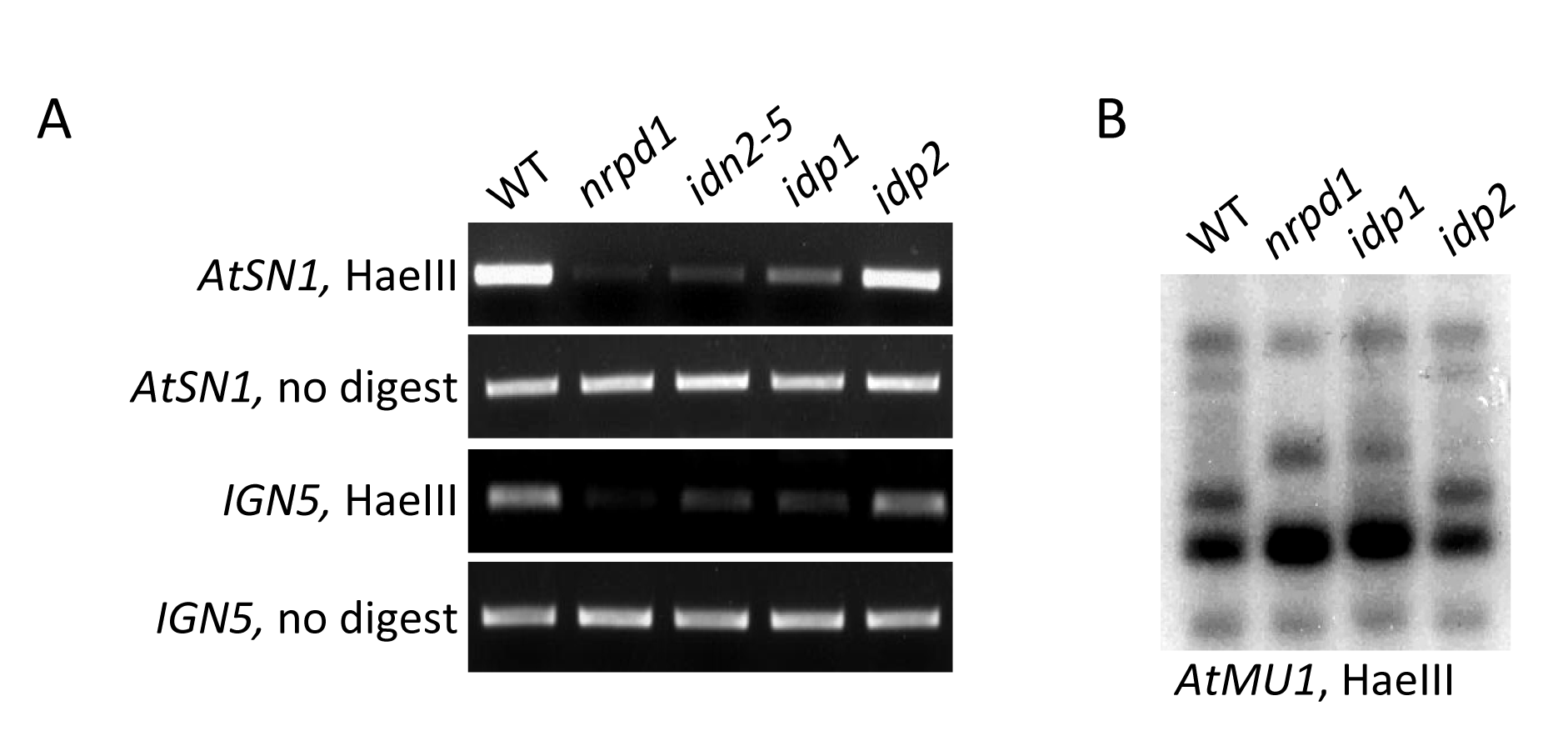

Supplement: Figure S4 — Effect of idp1 and idp2 on DNA methylation. (A) Effect of idp1 and idp2 on the DNA methylation level of AtSN1 and IGN5 was determined by chop-PCR. Genomic DNA was digested by the DNA methylation-sensitive restriction enzyme HaeIII, followed by amplification. (B) Effect of idp1 and idp2 on AtMU1 DNA methylation was tested by southern blotting. Genomic DNA was digested by HaeIII followed by southern hybridization. (TIF) [file pgen.1002693.s004.tif]

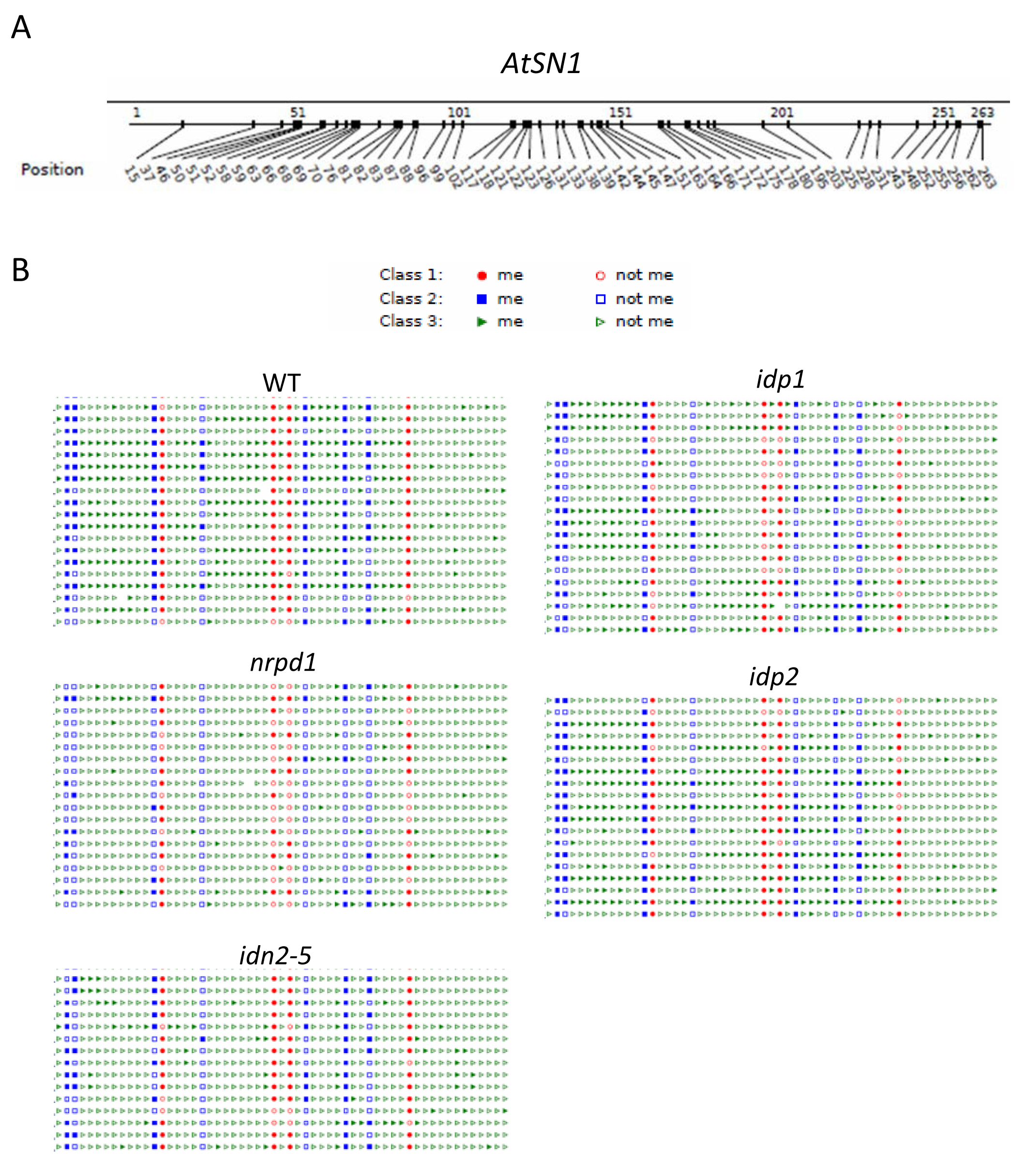

Supplement: Figure S5 — Diagram of the bisulfite sequencing results of AtSN1. The bisulfite sequencing results were analyzed by CyMATE. (A) The positions of all cytosines in the tested AtSN1 sequence. (B) The methylation status of all cytosines for each clone in WT, nrpd1, idn2-5, idp1, and idp2 is shown. The cytosine methylation in different contexts (CG, CHG, and CHH) is diagramed as indicated. Each line represents the cytosine methylation status for each clone. Class 1, Class 2, and Class 3 represent the cytosines at CG, CHG, and CHH sites, respectively. “H” is A, T, or C. (TIF) [file pgen.1002693.s005.tif]

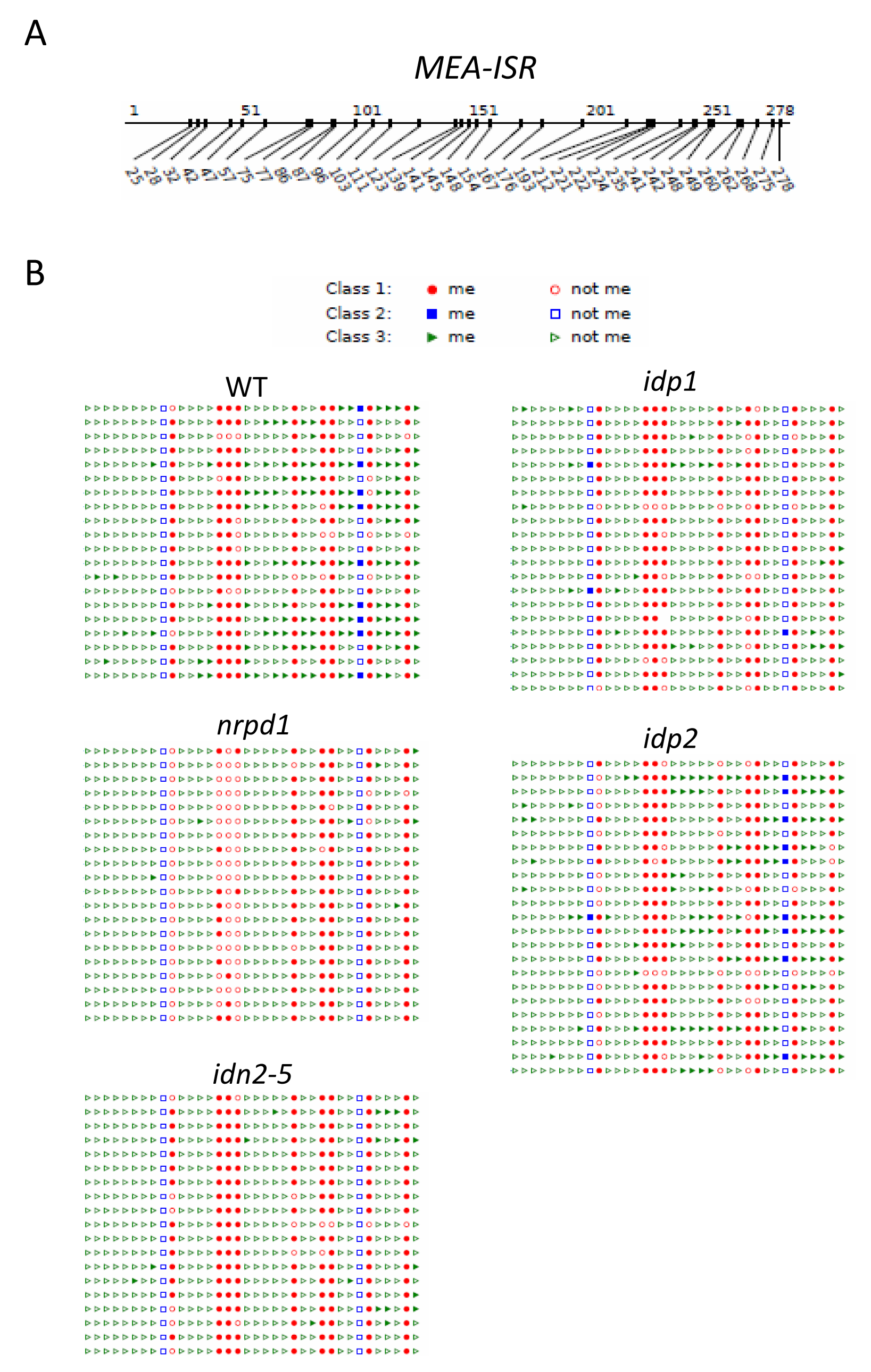

Supplement: Figure S6 — Diagram of the bisulfite sequencing results of MEA-ISR. (TIF) [file pgen.1002693.s006.tif]

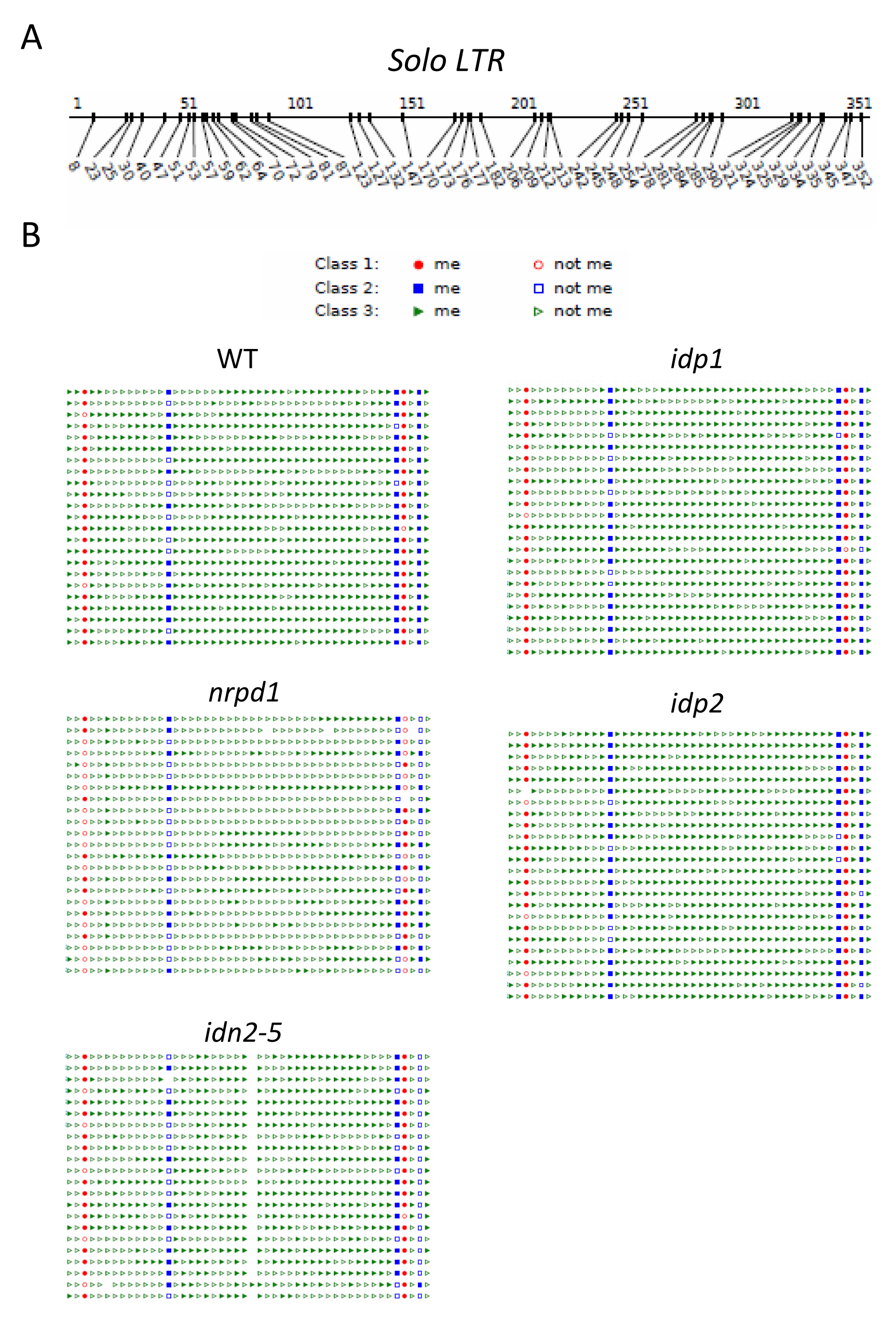

Supplement: Figure S7 — Diagram of the bisulfite sequencing results of Solo LTR. (TIF) [file pgen.1002693.s007.tif]

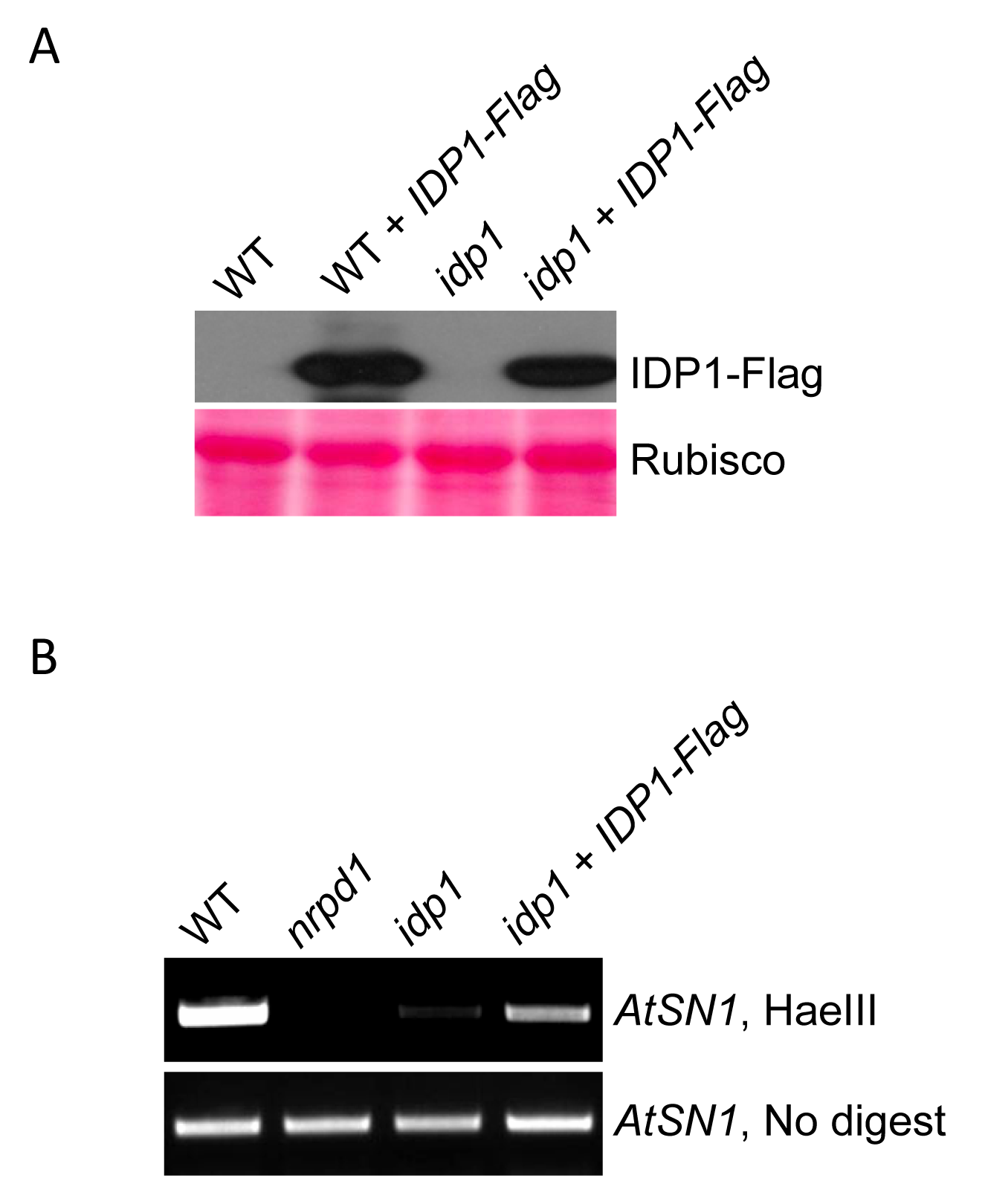

Supplement: Figure S8 — The IDP1-3xFlag transgene is functional in vivo. (A) The expression of the IDP1-3xFlag transgene in wild type and idp1 was tested by the Flag antibody. Ponceau S staining of Rubisco is shown as a loading control. (B) The IDP1-3xFlag transgene complements the DNA methylation defect of AtSN1. AtSN1 methylation was tested by chop-PCR. (TIF) [file pgen.1002693.s008.tif]

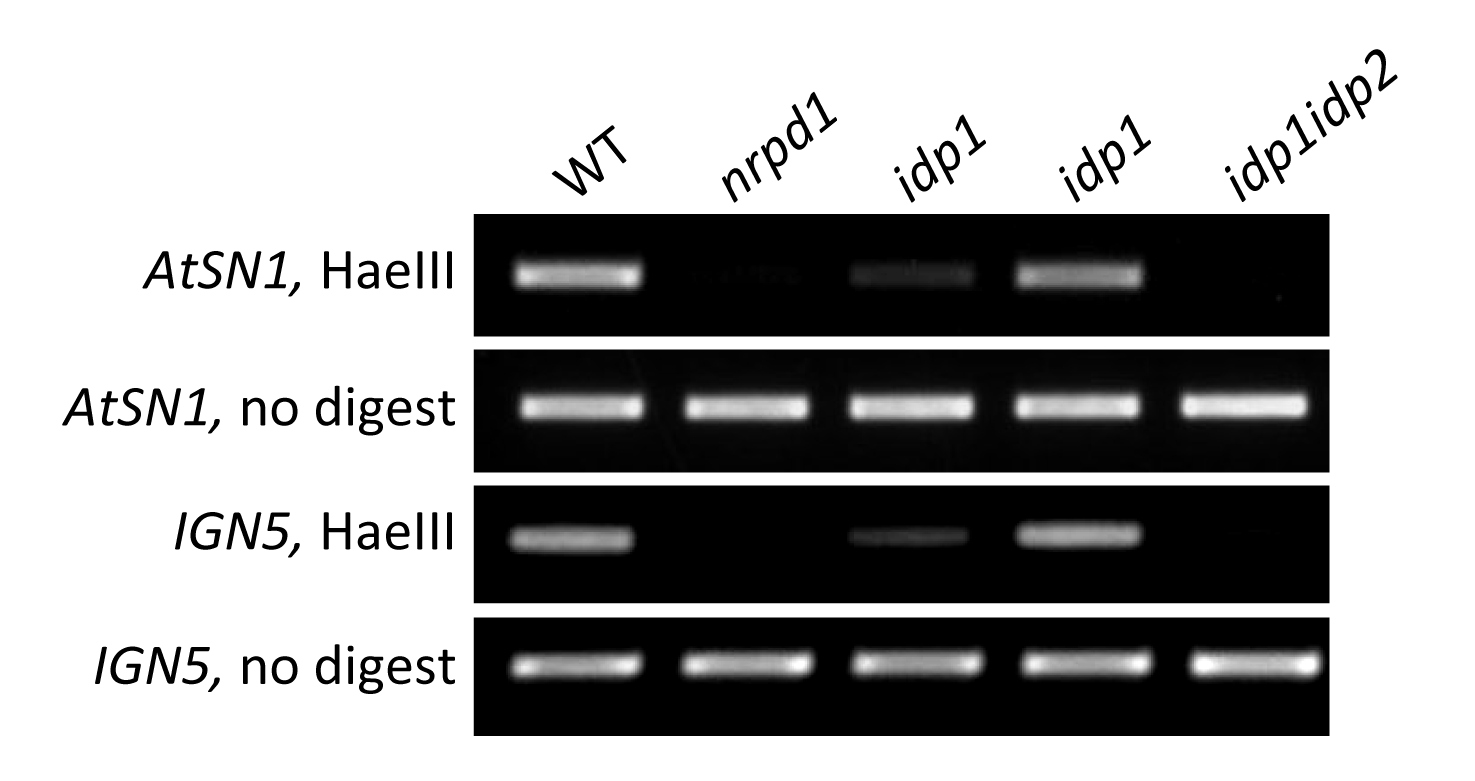

Supplement: Figure S9 — Detection of DNA methylation in the idp1idp2 double mutant. The DNA methylation level of AtSN1 and IGN5 was determined by chop-PCR. Genomic DNA from each indicated genotype was digested by the DNA methylation-sensitive restriction enzyme HaeIII, followed by amplification. (TIF) [file pgen.1002693.s009.tif]

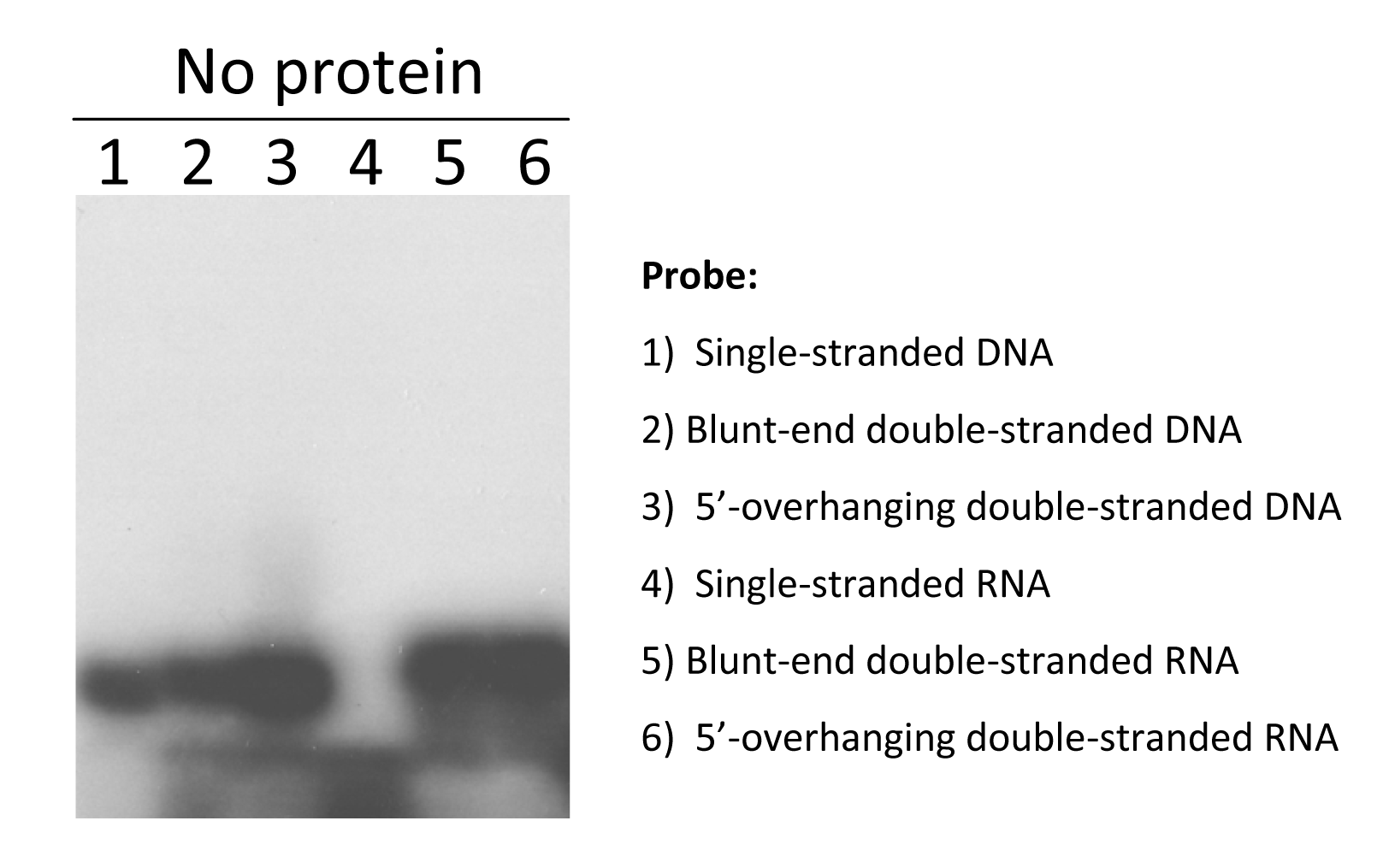

Supplement: Figure S10 — Each labeled nucleic acid was run on native gel when no protein was added. The result indicated that all nucleic acids were well labeled and purified. The identifications of probes (1–6) were shown in Figure 6B. (TIF) [file pgen.1002693.s010.tif]

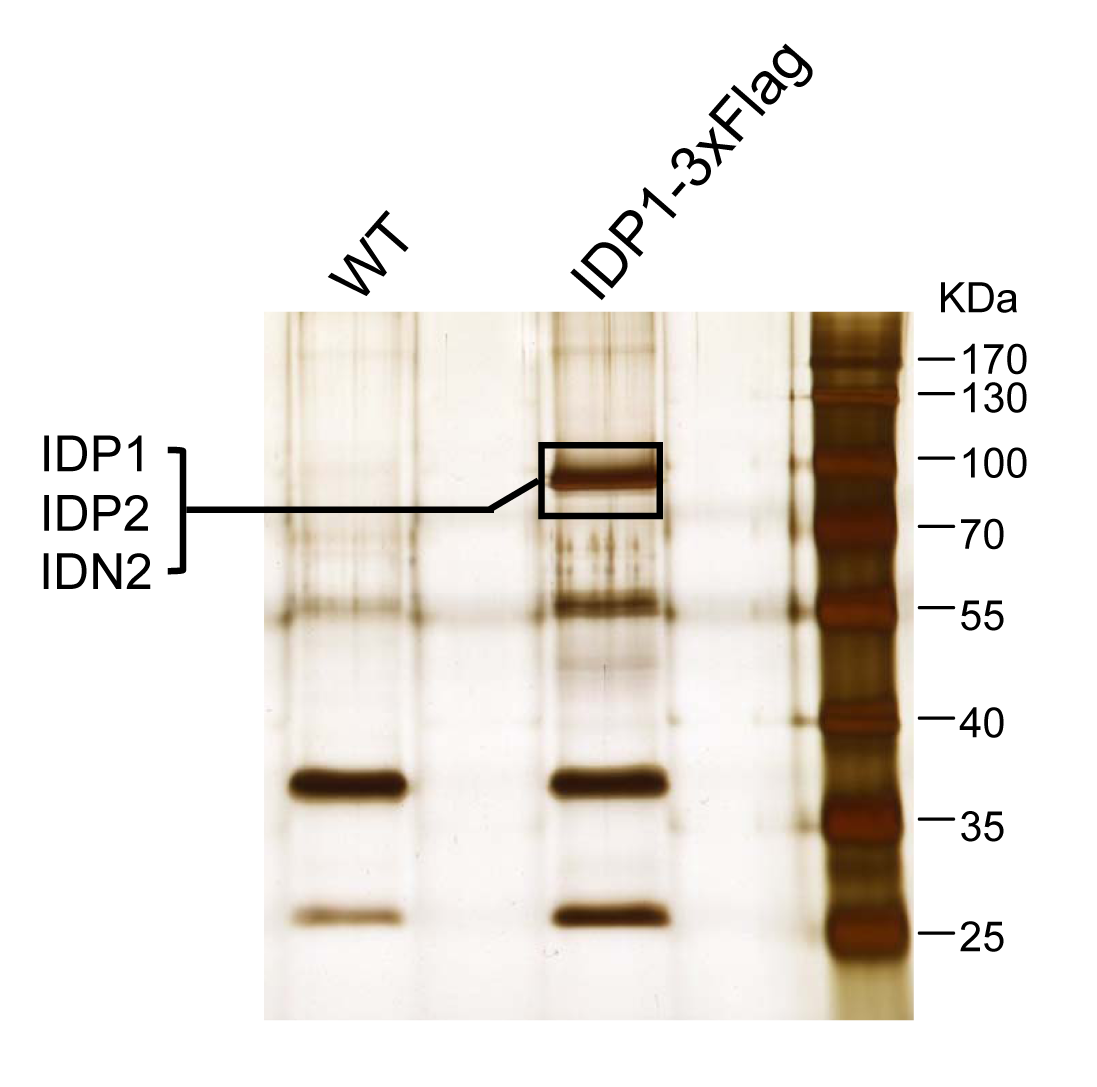

Supplement: Figure S11 — Co-purified proteins by IDP1-3xFlag pull down. Protein extracts were isolated from the IDP1-3xFlag transgenic plants as well as from the wild-type control. The extracts were subjected to affinity purification of IDP1-3xFlag by the Flag antibody. Total purified proteins were visualized by silver staining on SDS-PAGE gel. (TIF) [file pgen.1002693.s011.tif]

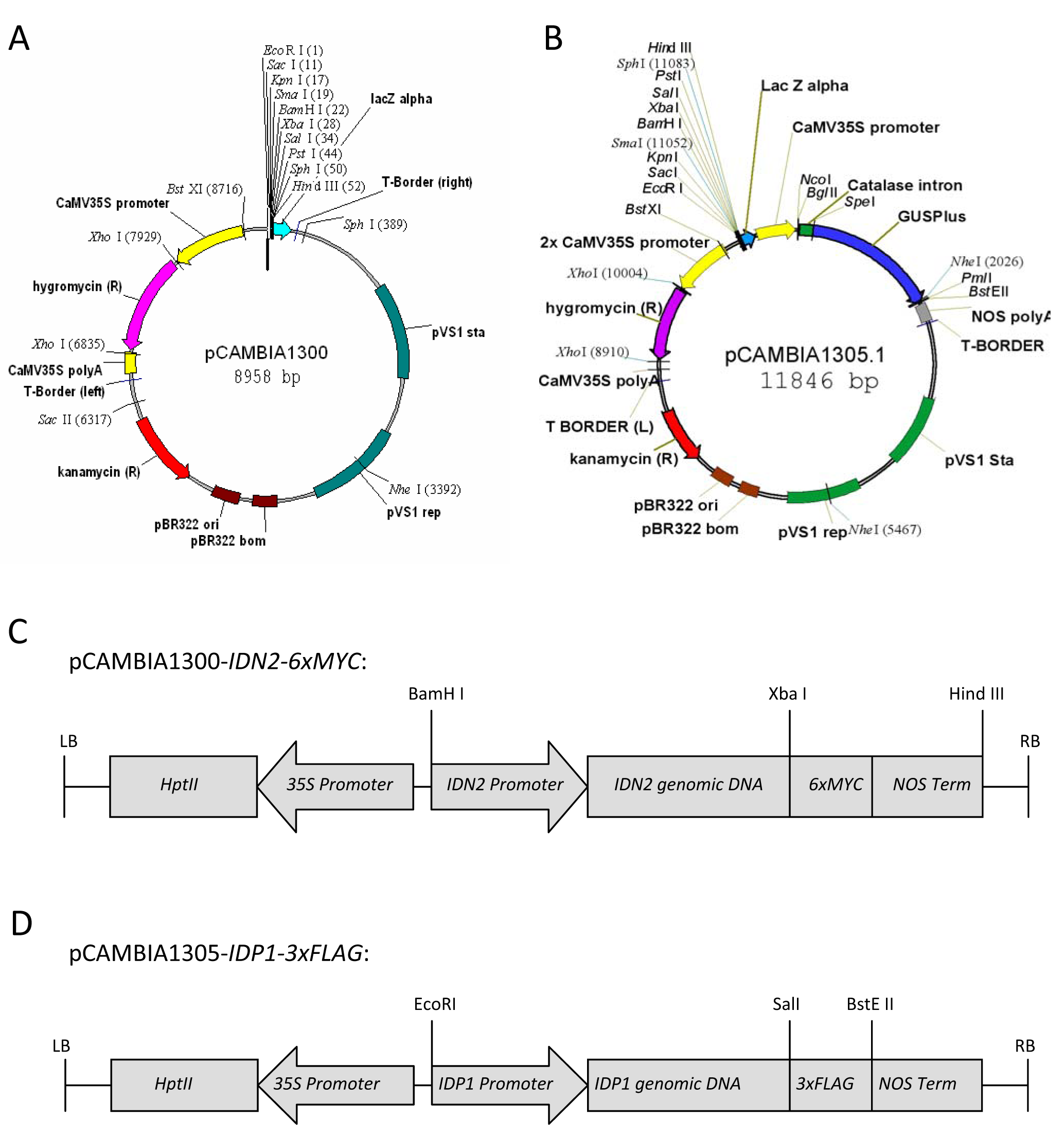

Supplement: Figure S12 — Diagram of the IDN2-6xMyc and IDP1-3xFlag constructs. (A, B) The backbones of the IDN2-6xMyc and IDP1-3xFlag constructs are pCAMBIA1300 and pCAMBIA1305, respectively. (C) Diagram of the IDN2-6xMyc construct. A 6xMyc encoding DNA sequence and a NOS terminator were inserted between XbaI and HindIII restriction sites. The native promoter-driven IDN2 genomic sequence was cloned in frame with the 6xMyc. (D) Diagram of the IDP1-3xFlag construct. The 35S promoter-driven GUS reporter was replaced by a 3xFlag encoding sequence between HindIII and BstEII restriction sites. The native promoter-driven IDP1 genomic sequence was cloned in frame with the 3xFlag between EcoRI and SalI sites. (TIF) [file pgen.1002693.s012.tif]
