# Supplementary material for: IDN2 and Its Paralogs Form a Complex Required for RNA–Directed DNA Methylation
Source: PLoS Genet. 2012 May 3;8(5):e1002693. doi: 10.1371/journal.pgen.1002693 (PMC3342958; doi:10.1371/journal.pgen.1002693)
Supplement: Table S2 — Mass-spectrometric analysis of IDP1-3xFlag affinity purification. Total protein extracts were isolated from the IDP1-3xFlag transgenic plants in the wild-type background and subjected to affinity purification of IDP1-3xFlag. The co-purified proteins were purified and used for mass-spectrometric analysis. Mascot score of each protein is shown. 1) The number is the total number of mass spectra matched to the corresponding protein, including the redundant ones that match to the same peptides. 2) The number is deduced from the “Matched queries” by removing the redundant peptides. 3) The number is calculated from “Matched queries” by removing the overlapped peptide sequences among its homologous protein family. (DOC) [file pgen.1002693.s014.doc]

**Table S2.** Mass-spectrometric analysis of IDP1-3xFlag affinity purification.

| Accession number | Protein | Mascot score | MW (Da) | ^1^Matched queries | ^2^Matched peptides | ^3^Unique matches |
| --- | --- | --- | --- | --- | --- | --- |
| IPI00523067 | AT1G15910 IDP1 | 8915 | 72590 | 269 | 61 | 189 |
| IPI00542085 | AT4G00380 IDP2 | 1311 | 73119 | 90 | 22 | 10 |
| IPI00524938 | AT3G48670 IDN2 | 143 | 74778 | 5 | 5 | 5 |
