# Supplementary material for: IDN2 and Its Paralogs Form a Complex Required for RNA–Directed DNA Methylation
Source: PLoS Genet. 2012 May 3;8(5):e1002693. doi: 10.1371/journal.pgen.1002693 (PMC3342958; doi:10.1371/journal.pgen.1002693)
Supplement: Table S3 — Summary of bisulfite sequencing results. The bisulfite sequencing results for AtSN1, MEA-ISR, and Solo LTR is summarized. The number of analyzed clones for each genotype and the number of each type of cytosine contexts are shown. The percentage of methylated cytosines for each cytosine context is indicated for each genotype. The numbers of methylated cytosines and total cytosines for each type of cytosine contexts are shown as “methylated cytosines/total cytosines” in parentheses. (DOC) [file pgen.1002693.s015.doc]

**Table S3.** Summary of bisulfite sequencing results.

|  |  | Cytosine methylation (%) | | | | |
| --- | --- | --- | --- | --- | --- | --- |
|  |  | WT | *nrpd1* | *idn2-5* | *idp1* | *idp2* |
| *AtSN1* | Clones | 19 | 19 | 15 | 20 | 19 |
|  | CG(4) | 92.1% | 52.6% | 83.3% | 72.5% | 84.2% |
|  |  | (70/76) | (40/76) | (50/60) | (58/80) | (64/76) |
|  | CHG(7) | 72.2% | 18.0% | 50.5% | 53.6% | 66.2% |
|  |  | (96/133) | (24/133) | (53/105) | (75/140) | (88/133) |
|  | CHH(46) | 29.9% | 5.1% | 8.4% | 17.2% | 26.2% |
|  |  | (261/874) | (45/874) | (58/690) | (158/920) | (229/874) |
| *MEA-ISR* | Clones | 20 | 20 | 19 | 21 | 23 |
|  | CG(9) | 87.2% | 58.9% | 90.6% | 87.8% | 83.1% |
|  |  | (157/180) | (106/180) | (155/171) | (166/189) | (172/207) |
|  | CHG(2) | 22.5% | 0.0% | 0.0% | 7.1% | 21.7% |
|  |  | (9/40) | (0/40) | (0/38) | (3/42) | (10/46) |
|  | CHH(25) | 24.2% | 1.4% | 3.8% | 4.8% | 19.7% |
|  |  | (121/500) | (7/500) | (18/475) | (25/525) | (113/575) |
| *Solo LTR* | Clones | 23 | 23 | 22 | 24 | 24 |
|  | CG(2) | 93.5% | 43.5% | 86.4% | 95.8% | 91.7% |
|  |  | (43/46) | (20/46) | (38/44) | (46/48) | (44/48) |
|  | CHG(3) | 85.5% | 47.8% | 39.4% | 90.3% | 88.9% |
|  |  | (59/69) | (33/69) | (26/66) | (65/72) | (64/72) |
|  | CHH(43) | 71.6% | 23.8% | 51.5% | 70.2% | 71.9% |
|  |  | (708/989) | (235/989) | (487/946) | (724/1032) | (742/1032) |
